# Uncovering patterns of white matter degeneration in normal aging: Links between morphometry and microstructure

**DOI:** 10.1162/imag_a_00247

**Published:** 2024-08-02

**Authors:** Tyler D. Robinson, Yutong L. Sun, Paul T.H. Chang, J. Jean Chen

**Affiliations:** Rotman Research Institute, Baycrest, Toronto, Canada; Medical Biophysics, University of Toronto, Toronto, Canada; Biomedical Engineering, University of Toronto, Toronto, Canada

**Keywords:** white matter, diffusion, aging, microstructure, morphometry, tractography

## Abstract

While tract-wise differences in volume and microstructure are common targets of investigation in age-related changes in the white matter (WM), there has been relatively little exploration into other attributes of tract morphometry or its relation to microstructure in vivo, and limited understanding on how they jointly inform the understanding of the WM aging trajectory. This study examines 10 WM tracts for tract-wise differences in morphometry (i.e., volume, length, and volume-to-length ratio) and microstructural integrity (i.e., fractional anisotropy, mean diffusivity, axial diffusivity, and radial diffusivity) using diffusion MRI data from the Human Connectome Project in Aging (HCP-A) with the goal of laying the foundation for a more comprehensive model of age-related WM microstructure-morphometry trajectories with a special focus on age-shifted correlations and sex differences. Results indicated that degeneration in microstructure was detectable at younger ages than changes in morphometry, with widely heterogeneous patterns of interrelation and morphometry-microstructural associations in aging both across tracts and between sexes. Multi-parametric signatures of decline suggest differing stages or mechanisms of degeneration across tracts, with female subjects exhibiting a higher proportion of tracts in later stages of decline than males. This work highlights the value of integrating microstructural and morphometric measures of WM health, and encourages the integration of yet more modalities in improving our mechanistic understanding of WM aging.

## 1 Introduction

Changes in function, structure, and microstructure in the human brain occur throughout the adult lifespan (Baker et al., 2014;Bartzokis, 2004;Bookheimer et al., 2019;Buyanova & Arsalidou, 2021;Chad et al., 2018;Kiely et al., 2022;Kodiweera et al., 2016;Piguet et al., 2009). Earlier prediction of regional declines is invaluable to medical intervention for the postponement or reversal of progressive cognitive and structural decline (de Groot et al., 2015). To this end, evidence suggests that early microstructural degeneration can be observed in the white matter (WM) prior to their emergence in cortical regions (Debette & Markus, 2010;Hu et al., 2021;Madden et al., 2012;Seiler et al., 2018). Consistent changes in WM macro-/microstructure and histology have been demonstrated in advancing age (Baker et al., 2014;Bastin et al., 2010;Choy et al., 2020;Ouyang et al., 2021;Tang et al., 1997), with overall volume decreases identified in normal aging (Bastin et al., 2010;Piguet et al., 2009;Tang et al., 1997). Notably, WM volume decreases by up to 28% in adult aging (Pakkenberg & Gundersen, 1997), but there is still a limited understanding of the microstructural variations underlying this volume reduction. In order to understand the mechanisms underlying WM decline, as well as how they may vary across the brain, it is important to incorporate knowledge of age-related changes in both WM morphometry and microstructure (Bartzokis, 2004;Benitez et al., 2018;Maffei et al., 2021;Yendiki et al., 2011). Building a more comprehensive modeling of age-related WM changes offers an invaluable tool to detect degeneration at earlier stages.

While WM volume loss in aging has been established in neuroimaging literature, the regional distribution of this volume loss is as of yet under-defined. Decreases in cumulative fiber length (i.e., the total length across the brain as extrapolated from histological samples) have been observed in both humans and animals using diffusion MRI and microscopy, respectively (Baker et al., 2017;Marner et al., 2003). This loss can, in part, be attributed to the preferential culling of vulnerable axons, as fibers with lower axon diameter and less dense myelin sheaths are notably more vulnerable to this process (Tang et al., 1997). This further suggests that a significant contributor to WM morphometrical losses in aging may be a reduction in the total number of fibers and a concomitant increase in mean axon diameter in surviving tissue, as opposed to changes in the length of surviving axons. As WM fibers with higher diameter are also typically longer (Liewald et al., 2014), aging tracts should demonstrate increases in the length-to-volume ratio as shorter, narrower fibers are culled and the larger surviving fibers comprise a greater portion of the WM tissue. However, to what degree this potential effect contributes to changes in WM morphometry, considering the general atrophy of brain volume with age (Azevedo et al., 2019;Fjell et al., 2009,^2014^;Sala et al., 2012), is unclear. Individual fiber tracts in the white matter exhibit varying degrees of vulnerability to age-related fiber culling (Choy et al., 2020). Differences in mean regional fiber composition before and over the course of degeneration may then account for previous observations of both decreasing and increasing mean fiber length in specific major white matter bundles, including the splenium and superior longitudinal fasciculus (Ouyang et al., 2021). This calls into question the assumption that overall decreases in fiber length correspond to regional decreases in tract length. Alternate mechanisms of shortening in measurable fiber length, such as damage and delayed clearing of remaining debris (Avellino et al., 1995) or the presence of early-stage degenerative conditions targeting short-range structural connections (Wu et al., 2022), may also influence measures of mean tract length. Morphometrical research has noted cross-sectional tract-wise differences in age-related decline with regards to volume and microstructure (Bastin et al., 2010;Choy et al., 2020), but fiber length has received considerably less attention in aging despite evidence of distinct contributions to network organization (Bajada et al., 2019).

The tools with which living WM can be accurately delineated into discrete tracts using imaging data are both relatively new and continually improving (Maffei et al., 2021), but there have already been several recent studies of tract-wise age-related effects in the WM (Beck et al., 2021;Cox et al., 2016;Schilling et al., 2022). These papers generally demonstrate that WM aging is tract specific, as will be detailed later in this work. Nonetheless, as many existing works do not address potential sex differences, distinctions between sex-specific trajectories of decline remain unclear. The presence of hormonal differences in aging, in particular the impact of menopause and its observed effects on microstructural integrity (Mosconi et al., 2021), suggest that there may be similar variation in morphometric declines between sexes. Understanding the degree to which microstructural and morphometric declines are interrelated is essential to disentangling both variations in regional WM aging across the brain, and sex differences in WM aging more generally.

Reported to show alterations earlier than cortical morphometry (Debette & Markus, 2010;Hu et al., 2021;Madden et al., 2012;Seiler et al., 2018), diffusion MRI metrics such as fractional anisotropy (FA) and mean diffusivity (MD) offer proxy measures for WM microstructural integrity (Madden et al., 2012;Stadlbauer et al., 2008). Phenomena such as culling of axonal fibers and demyelination of surviving axons result in measurable changes in diffusion, such that mean diffusivity increases and fractional anisotropy decreases as axon bundles become less dense (Choy et al., 2020;Fan et al., 2019;Kodiweera et al., 2016). Existing research has also identified heterogeneous rates of decline in different WM regions, such as sections of the corpus callosum, and broader anterior and posterior regions of the brain (C. L. Armstrong et al., 2004;Brickman et al., 2012;Fan et al., 2019;Kiely et al., 2022;Seiler et al., 2018). Declines in microstructural integrity in aging WM are associated with both WM hyperintensities and implicated in declining cognitive performance (Debette & Markus, 2010;Hu et al., 2021). This knowledge is of considerable interest for anticipating pathological changes and prioritizing interventions (Debette & Markus, 2010). Tract-wise studies and inter-tract comparisons of WM microstructural variations in aging remain limited, with fewer still addressing sex differences on a tract-wise basis. Recent work bySchilling et al. (2022)involved the largest data set and number of tracts to date, and highlighted the heterogeneity in multiple morphometric dimensions across the WM, which is a strong motivation for this study.

We reasonably expect microstructural variations to underlie morphometrical variations in aging (N. M. Armstrong et al., 2020;Hoagey et al., 2019;Taki et al., 2011), but interaction between morphometrical and microstructural declines represents a sparsely addressed topic (Bastin et al., 2010;Kezele et al., 2008;Seiler et al., 2018). While previous work has established the use of multivariate modeling to investigate the relationship between grey-matter and WM aging (Hoagey et al., 2019), in the WM per se, microstructural and morphometric age variations are largely assessed independently (Sala et al., 2012;Schilling et al., 2022). Prior investigation notes a decrease in overall fiber count with age that could account for measurable changes in WM volume (Marner et al., 2003). This would pose microstructural declines as a direct cause of morphometrical changes in volume, though whether these changes are contemporaneous is uncertain. However, histological findings in animals and humans also note that surviving WM fibers are thicker with advancing age (Fan et al., 2019,^2020^;Stahon et al., 2016), potentially related to lower fiber density in WM tracts (Choy et al., 2020;Fan et al., 2019;Kiely et al., 2022;Marner et al., 2003). However, changes in density may not necessitate a uniform decrease in volume, as space within fiber bundles is occupied by intercellular fluid (Kodiweera et al., 2016). Uncertainty in these microstructural-morphometric relationships make it difficult to anticipate the relationships between microstructural decline and changing WM morphometry with age. Given known baseline differences between WM regions, such as fiber composition (Huang et al., 2020), perfusion (Robinson et al., 2023), and myelin architecture (Groeschel et al., 2016), we propose that the time has come to build a model of WM aging that unites these multiple aspects of WM aging.

To that end, this study sought to map interactions in tract-specific WM morphometry and microstructure variations in aging through a lens of sex dependence. Tract-wise MD and FA, as well as axial diffusivity (AD) and radial diffusivity (RD) are used as the target microstructural metrics of this study. The morphometry of each tract is quantified in measures of tract-wise mean length, volume, and volume-length ratio, and these are, in turn, compared to the microstructural measures. We hypothesize that: (1) age-related changes in microstructure will be detectable earlier than changes in morphometry; that (2) due to the potential causality between microstructural and morphometric variations, age-related microstructural differences will be greater in tracts that demonstrate greater age-related morphometrical differences; that (3) relationships between age-related changes in morphometry and microstructure will differ by sex; and (4) given patterns of preferential WM fiber culling (Marner et al., 2003;Tang et al., 1997) and associations between axon diameter and axon length (Liewald et al., 2014), there would be interactions between age-related differences in fiber length and volume, specifically, that tracts that become thinner with age will also have higher mean tract lengths.

## 2 Methods

### 2.1 Subjects and data acquisition

This study included data from 535 healthy adult subjects (300 female, aged 36–100 years, approx. 48% postmenopausal based on age) of the Human Connectome Project in Aging (HCP-A) dataset (OMB Control# 0925-0667) (Bookheimer et al., 2019;Harms et al., 2018). All subjects were in generally good health and without pathological cognitive impairment (i.e., stroke, clinical dementia) and provided informed consent for their inclusion in the HCP-A dataset, which is freely available under appropriate data usage agreements. Following manual quality control, 32 subjects were excluded due to MRI artifacts. An additional three subjects (100 years of age) were excluded as age outliers due to a lack of subjects in the 90–100 age range. This resulted in a final sample size of 500 subjects (294 female, aged 36–89). Subject age distribution and education are reported inTable 1. Moreover, we determined hypertension status based on mean-arterial pressure (Kandil et al., 2023), identifying approximately 25% of all subjects potentially qualifiable for Stage 1 hypertension.

**Table 1.** Sample demographics.

	Count	Mean age (years)	Median age (years)	Age range (years)	Age Stdev (years)	Hypertension count	Education (years)
All	500	57.88	56	36–89	14.3	133	17.41
Female	294	57.27	54	36–89	14.4	63	17.29
Male	206	58.76	58	36–89	14.2	70	17.58

Demographic statistics for all subjects, female subjects, and male subjects. All subjects were in good health and without pathological cognitive impairment (i.e., stroke, clinical dementia).

Each data set included a whole-brain T1-weighted structural MRI (0.8 mm isotropic resolution) and diffusion-weighted MRI (dMRI), collected on one of four matched Siemens Prisma 3T MRI scanners, with a 1.5 mm isotropic voxel resolution, MB = 4, with 93 directions at b = 1500 s/mm^2^. Further details on the structural and diffusion image acquisition can be found inHarms et al. (2018).

### 2.2 Image analysis

**Fig. 1.**
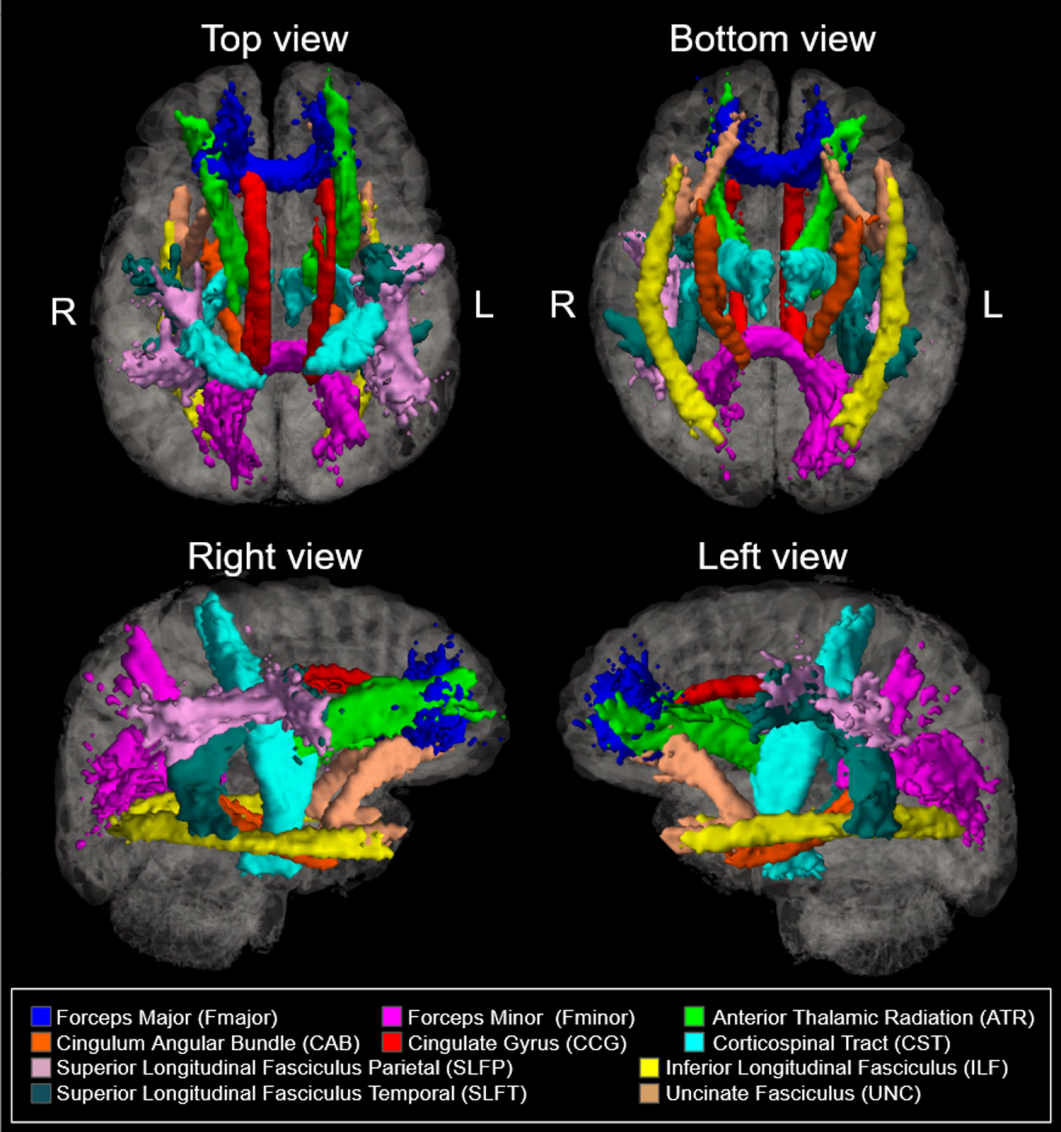
Ten bilateral tracts of interest reconstructed using FreeSurfer TRACULA.

dMRI data were corrected for eddy-current and susceptibility-related distortions via EDDY prior to tract reconstruction. Eighteen major WM tracts were reconstructed from local T1 maps using the Tracts Constrained by Underlying Anatomy (TRACULA) tool in Freesurfer version 7.2.0 (Maffei et al., 2021;Yendiki et al., 2011). Tracts reconstructed by TRACULA’s trac-all command represent pathways in which each adjacent voxel in a given path has a 99% probability of containing the next fiber in said path based on diffusion characteristics to ensure the highest level of anatomical likelihood while excluding potential WM hyperintensities. All values below 20% of the maximum posterior probability distribution were thresholded prior to extracting diffusion values. The TRACULA pipeline additionally includes correction for eddy current distortion, brain extraction, tensor calculation, and affine registration. Per-subject DTI images were registered in local space using Freesurfer’s TKRegister function, and FMRIB Software Library version 6.0.5 (FSL)’s fslmaths function was then used to produce per-subject masks of each WM tract in local space. The 18 reconstructed tracts were collapsed bilaterally to produce 10 tracts of interest: the major forceps (Fmajor), minor forceps (Fminor), anterior thalamic radiation (ATR), cingulum angular bundle (CAB), cingulate gyrus (CCG), corticospinal tract (CST), inferior longitudinal fasciculus (ILF), superior longitudinal fasciculus-parietal (SLFP), superior longitudinal fasciculus-emporal (SLFT), and the uncinate fasciculus (UNC) (Fig. 1). In single-tract analyses, Rosner’s tests were performed to identify and exclude volume outliers per tract. The resulting subject counts were as follows: Fmajor (N = 493), Fminor (N = 493), ATR (N = 497), CAB (N = 490), CCG (N = 495), CST (N = 491), ILF (N = 497), SLFP (N = 497), SLFT (N = 495), and UNC (N = 495).

#### 2.2.1 Morphometry

From these segmented regions, the morphometric measures calculated include tract volume, length, and volume-to-length ratio (VLR) using Python (Python Software Foundation) along with fslmaths (FMRIB Software Library version 6.0.5 (FSL)). We computed both raw morphometrics and baseline (young)-normalized age-related fractional variations.

1. Tract volume (mm^3^):
  - Raw tract volume (“Volume”): calculated as the total number of voxels (1.5 x 1.5 x 1.5 mm^3^) included in each masked tract region.
  - Percent tract volume change (“Volume_perc_”): calculated as individual raw tract volume divided by the mean raw tract volume of the youngest 10% of subjects, calculated separately for male and female subsets.
2. Tract length (mm):
  - Raw tract length (“Length”): calculated as the mean streamline length from 1500 streamlines per tract in TRACULA.
  - Percent tract length change (“Length_perc_”): calculated as individual raw tract length divided by the mean raw tract length of the youngest 10% of subjects, calculated separately for male and female subsets.
3. Tract volume-to-length ratio (VLR) (mm^2^):
  - Raw VLR (“VLR”): calculated as the ratio of tract volume to length. This represents the cross-sectional area of a tract.
  - Percent tract VLR change (“VLR_perc_”): calculated as individual raw tract VLR divided by the mean raw tract VLR of the youngest 10% of subjects, calculated separately for male and female subsets.

#### 2.2.2 Microstructure

Fractional anisotropy (FA), mean diffusivity MD (m^2^/s), axial diffusivity (AD) (m^2^/s), and radial diffusivity (RD) (m^2^/s) maps were derived from the dMRI data using Dipy’s DKI tool to provide kurtosis-corrected DTI metrics (given the high b-value used in the HCP-A dMRI acquisitions). Tract masks generated in the morphometric assessment were applied to the local space diffusion images using fslmaths to generate mean FA and mean diffusivity values for each tract of interest of each participant. As with morphometric measures, baseline-normalized values (“FA_perc_”, “MD_perc_”, “AD_perc_”, “RD_perc_”) were calculated for each microstructural measure to determine percent difference from the youngest 10% of the sample, calculated separately for male and female subsets.

### 2.3 Statistical analyses

#### 2.3.1 Morphometry

In these analyses, we first regressed age against morphometry in tract-wise linear and quadratic regressions (with age as a non-linear term), using both raw and baseline-normalized values. Additionally, we regressed both age and sex against tract-wise morphometry (Length_perc_and VLR_perc_) in multivariate linear regressions. In the multivariate models, baseline normalization was selected in favor of anatomical normalization methods to emphasize the fractional variations referenced to a young age. All analyses in this study were conducted in R (version 4.1.1). False detection rate (FDR) adjustment was applied to resulting*p*-values to correct for multiple comparisons for each hypothesis grouping, that is, results from within each pairing of microstructure-morphometry measures across tracts. Per-hemisphere analyses were conducted to verify the similarity of age effects between hemispheres (Supplementary Fig. 3). An additional model of age and sex effects with the inclusion of an age-sex interaction regressor was performed to verify the presence of significant sex differences in tract-wise age effects (Supplementary Table 2;Supplementary Fig. 4).

#### 2.3.2 Microstructure

Age-related microstructure differences were analyzed by first regressing age against tract-wise microstructural measures (MD_perc_and FA_perc_) in linear and quadratic regressions (with age as a non-linear term). Additionally, we regressed both age and sex against microstructural measures in tract-wise multivariate regressions. FDR adjustment was again applied to the resulting*p*-values. Likewise, baseline-normalized values were used in the multivariate regression. As with morphometric regressions, additional hemisphere-split age effects were conducted (Supplementary Fig. 3), along with analysis of age-sex interaction effects (Supplementary Table 2;Supplementary Fig. 4).

#### 2.3.3 Age-displaced analysis: Cross-correlation function (CCF)

To determine the temporal patterns in the age-associated correlations of morphometry and microstructure across age groups, an exploratory age-displacement analysis using cross-correlations functions (CCF) with baseline-normalized parameter values as inputs was performed using Matlab (Mathworks Inc., Natick, USA). In the absence of longitudinal data, cross-correlation between variables A and B provides an approximation for how A and B can be related on a cross-subject age-related trajectory, which can generate hypotheses to later be tested longitudinally. In this analysis, a positive peak-correlation shift indicates that variations in B are observable before they are observable in variable A. Only variables demonstrating significant non-linear age effects on a tract-wise basis were examined in this analysis, using a curvature threshold of 0.0005, where curvature was computed using Matlab’s*curvature*function. CCFs were calculated based on the models of age effects on six paired tract-wise microstructure/morphometry measures, along with cross-comparisons for AD_perc_/RD_perc_versus FA_perc_and Length_perc_versus Volume_perc_:

- Length/Microstructure: Length_perc_versus AD_perc_, Length_perc_versus RD_perc_, Length_perc_versus FA_perc_
- Volume/Microstructure: Volume_perc_versus AD_perc_, Volume_perc_versus RD_perc_, Volume_perc_versus FA_perc_
- AD_perc_versus FA_perc_
- RD_perc_versus FA_perc_
- Length_perc_versus Volume_perc_

#### 2.3.4 Morphometry versus microstructure

Multivariate regression was conducted to assess the relationships between morphometry, microstructure, age, and sex in our sample. This model was first applied to whole-WM Length_perc_and VLR_perc_values, then to the individual values for each tract of interest. Separate regressions were conducted for each pairing of morphometric and microstructural measures, that is: {FA_perc_, MD_perc_} = f(Length_perc_/VLR_perc_+ Age + Sex). We excluded Volume_perc_from the model as it would be redundant with the combination of Length_perc_and VLR_perc_, which each has a more specific dimensional interpretation The above analyses were conducted in R (version 4.1.1) with False Detection Rate (FDR) multiple comparison corrections applied on a per-model basis for all regressions.

#### 2.3.5 Multimodal associations: Canonical correlation analysis (CCA)

As a method for multimodal fusion, canonical correlation analysis (CCA) was conducted to examine the degrees to which multiple variables of interest in the morphometric and microstructural categories simultaneously contributed to morphometry-microstructure relationships across the lifespan. CCA allows the creation of linear combination vectors of multiple measures to examine the degree of contribution (or loading) of each constituent variable to the relationship between the tested categories. Two column vectors were computed using our morphometric (*V*_1_) variables (Length_perc_, Volume_perc_) and microstructural (*U*_1_) variables (AD_perc_, RD_perc_, FA_perc_) in Matlab. The linear combinations of*V*and*U*vectors which produced the highest correlation were then calculated to determine on a tract-wise basis which variables loaded most strongly to age differences by tract. This and following analyses substituted AD_perc_and RD_perc_for MD_perc_, as the AD_perc_and RD_perc_constituents of MD_perc_are more easily relatable to FA_perc_, and would render the inclusion of MD_perc_redundant. Values more than three scaled median absolute deviations from the median were excluded for each measure prior to CCA analysis. Split-half cross-validation was conducted through 10,000 permutations of random sampling of the male and female data sets. CCA was performed on each of the 10,000 randomly split samples, and mean and standard error of loadings associated with only statistically significant canonical correlations are plotted to confirm consistency of the CCA findings.

## 3 Results

### 3.1 Morphometry versus age and sex

Analysis of raw morphometric values identified significant sex differences in raw tract Length, Volume, and VLR for all 10 tracts when controlling for age effects. Male subjects demonstrated higher values in all three metrics across tracts. Following baseline normalization and controlling for age effects, significantly higher Length_perc_was identified in Fmajor and CCG for female subjects, as well as in ATR, ILF, SLFT, and UNC for male subjects. Female subjects demonstrated higher Volume_perc_and VLR_perc_in Fminor and CST, while men showed higher Volume_perc_and VLR_perc_in Fmajor, CAB, SLFP, SLFT, and UNC (Supplementary Table 1).

Initial analysis of relationships between age and raw morphometric measures identified significantly negative associations between age and raw tract Length in Fminor, CAB, CST, ILF, and SLFP,

SLFT and UNC. Significant negative associations between age and raw tract Volume were found in Fmajor, Fminor, ATR, CAB, CCG, ILF, SLFP, SLFT, and UNC, and between age and raw tract VLR in Fmajor, Fminor, ATR, CAB, CCG, ILF, SLFP, SLFT, and UNC. Following baseline normalization to produce percent values of each measure, morphometric values were regressed against age, with sex as a covariate of no interest. Linear and quadratic regression both resulted in significant associations (*p*< .05), although quadratic regression was found to provide a better fit (higher*r*^2^) than linear regression for age effects on Length_perc_in Fmajor, ATR, CAB, CCG, SLFP, and UNC. Similarly, quadratic, rather than linear declines provided better fits for age effects on Volume_perc_in ATR, CCG, ILF, and UNC, and age effects on VLR_perc_in Fmajor, Fminor, ATR, ILF, SLFP, SLFT, and UNC (Supplementary Fig. 1andSupplementary Table 3). Nonetheless, a summary of the age effects can be adequately approximated by the summary of linear slopes shown inFigure 2, with significant linear associations found between age and Length_perc_in Fminor, CAB, CCG, CST, ILF, SLFP, SLFT, and UNC and significant linear age association with Volume_perc_and VLR_perc_in all tracts. Overall, a higher slope for age effects in Volume_perc_, in comparison to Length_perc_, suggests a pattern of tract “thinning” across the WM, consistent with VLR_perc_declines in all tracts.

**Fig. 2.**
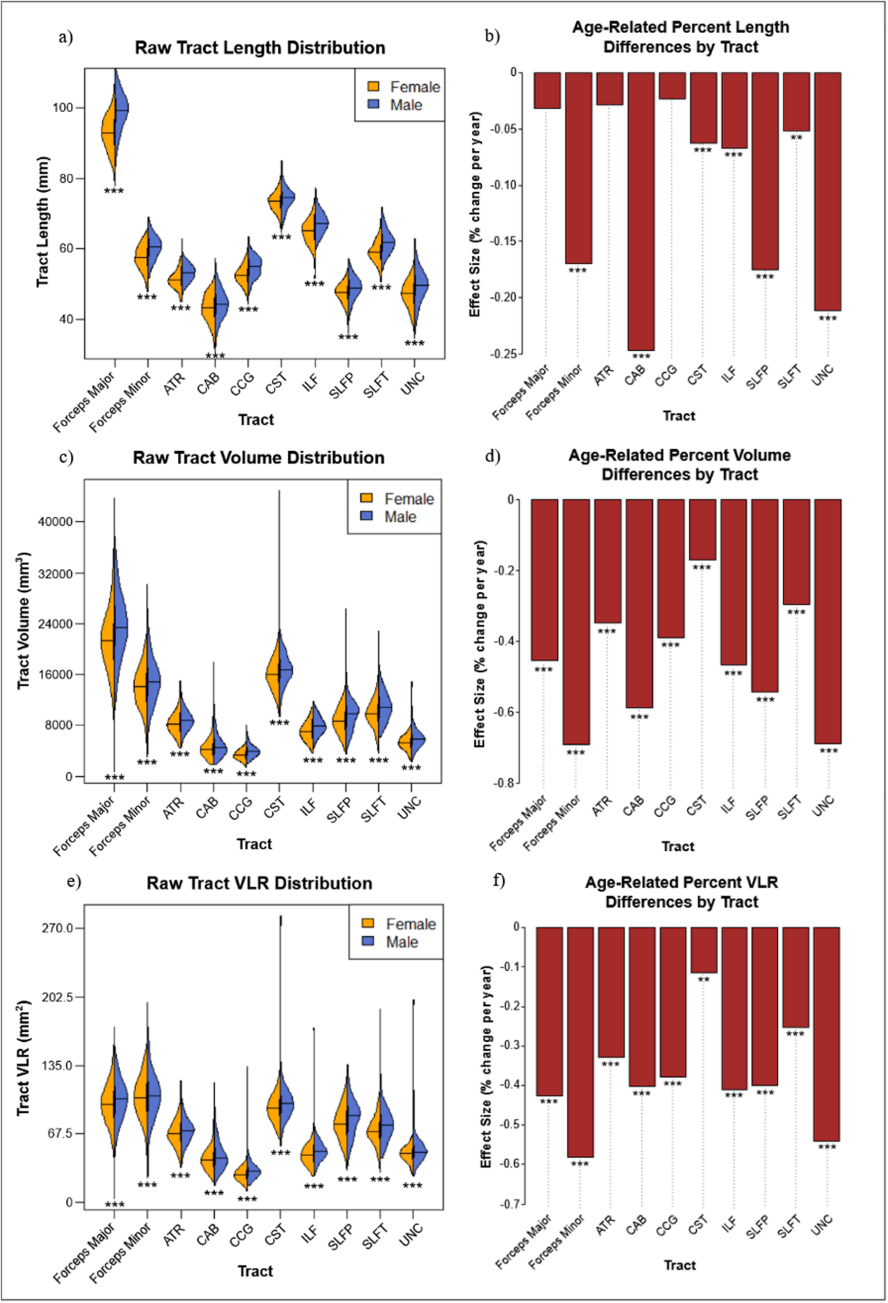
Tract-wise sex (a, c, e) differences on raw tract length (a), volume (c), and VLR (e) as well as age-related differences in Length_perc_(b), Volume_perc_(d), and VLR_perc_(f). Significant effects by sex and age are denoted with asterisks (***p< .001, **p< .01).

### 3.2 Microstructure versus age and sex

**Fig. 3.**
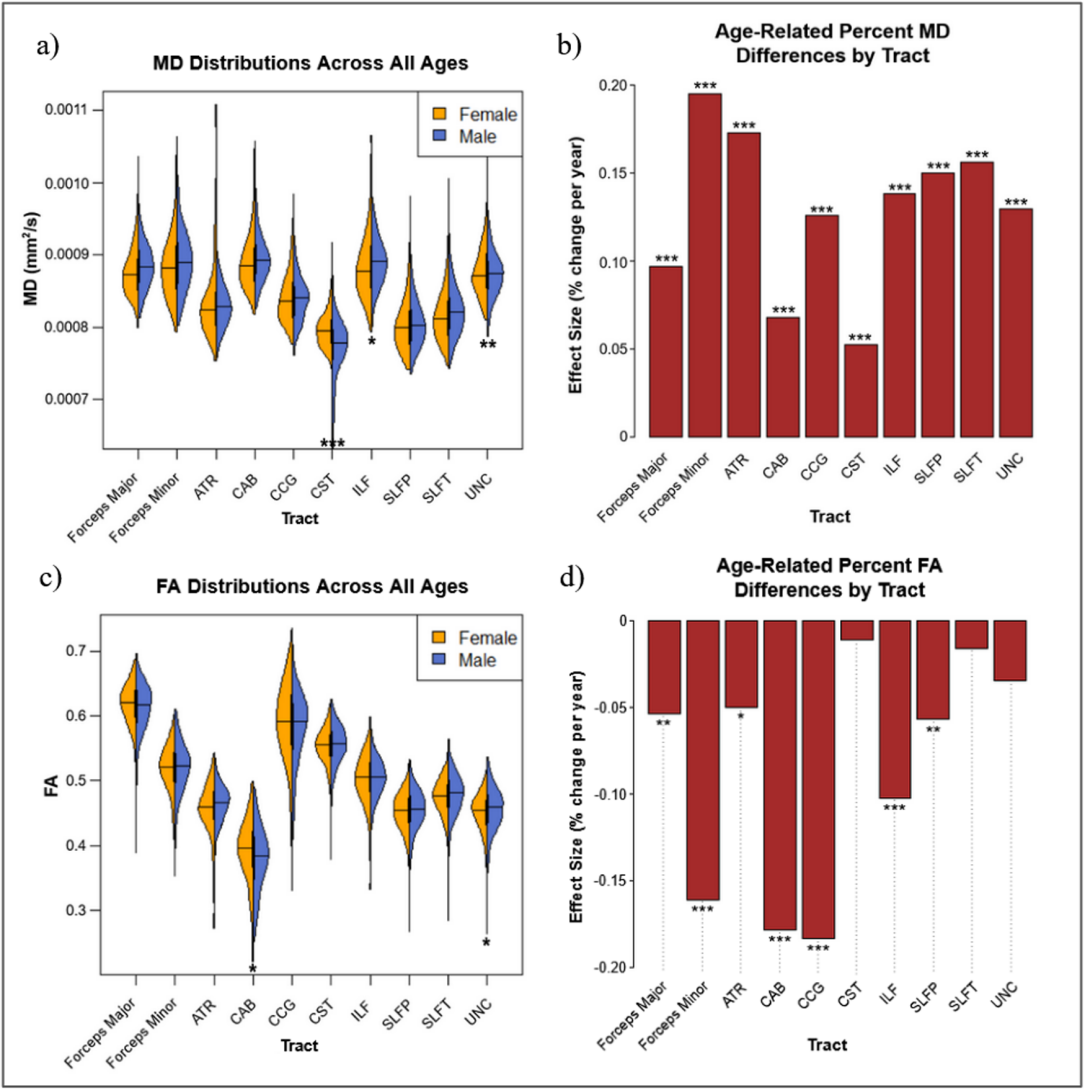
Raw MD and FA distributions separated by sex (a, c), and tract-wise age effects for MD_perc_and FA_perc_(b, d). Significant effects by sex and age are denoted with asterisks (***p< .001, **p< .01, *p< .05).

All 10 tracts of interest showed significant positive relationships between age and MD_perc_. Age was also negatively associated with FA_perc_in Fmajor, Fminor, ATR, CAB, CCG, ILF, and SLFP (Fig. 3). Quadratic, rather than linear regression provided a better fit for age effects on MD_perc_in all tracts with the exception of the CAB. In contrast, linear regression provided a better fit for FA_perc_in Fminor, ATR, and CCG (Supplemental Fig. 2).

Regarding sex differences, when controlling for age effects, female subjects demonstrated significantly higher MD_perc_in Fmajor, ATR, CST, ILF, SLFP, SLFT, and UNC. Female subjects also demonstrated significantly higher FA_perc_in Fmajor and UNC, while male subjects demonstrated significantly higher FA_perc_in Fminor, CCG, SLFP, and SLFT (Supplementary Table 1).

### 3.3 Tract-wise cross-correlations

The 0.0005 curvature threshold was not met for Length_perc_in Fmajor, CST, SLFT, and UNC; for Volume_perc_in Fminor; for FA_perc_in Fmajor, ATR, CAB, ILF, and SLFP; and for RD_perc_in Fmajor, CAB, CCG, CST, SLFT, and UNC. Significant microstructure-morphometry correlations were identified in the majority of the remaining comparisons. A significant correlation between Length_perc_and FA_perc_was identified in Fminor at zero correlational shift (time lag at the peak cross-correlation). Correlations between Length_perc_and FA_perc_were identified at zero correlational shift in the SLFT; negative correlational shift in Fminor, CCG, and SLFP such that AD_perc_differences were detectable at younger ages than Length_perc_; and positive shift in the ATR such that Length_perc_differences were detectable at younger ages than AD_perc_. Length_perc_and RD_perc_correlations were found at negative shift in Fminor, SLFP, and SLFT such that RD_perc_changes were detectable before Length_perc_, and at positive shift in the ATR such that Length_perc_changes were detectable before RD_perc_.

Correlations between Volume_perc_and FA_perc_were identified at zero correlational shift in SLFT and UNC, and at negative shift in the CST such that FA_perc_differences were detectable at younger ages than Volume_perc_. Volume and AD correlations were identified at zero shift in SLFP and UNC; at negative shift in Fmajor, ATR, CST, ILF, and SLFT such that AD_perc_changes were detectable earlier than Volume_perc_; and at positive shift in the CCG such that Volume_perc_changes were detectable earlier than AD_perc_. Correlations between Volume and RD were identified at zero shift in the ATR and at negative shift in ILF and SLFP such that RD_perc_changes were detectable at younger ages than Volume_perc_.

**Fig. 4.**
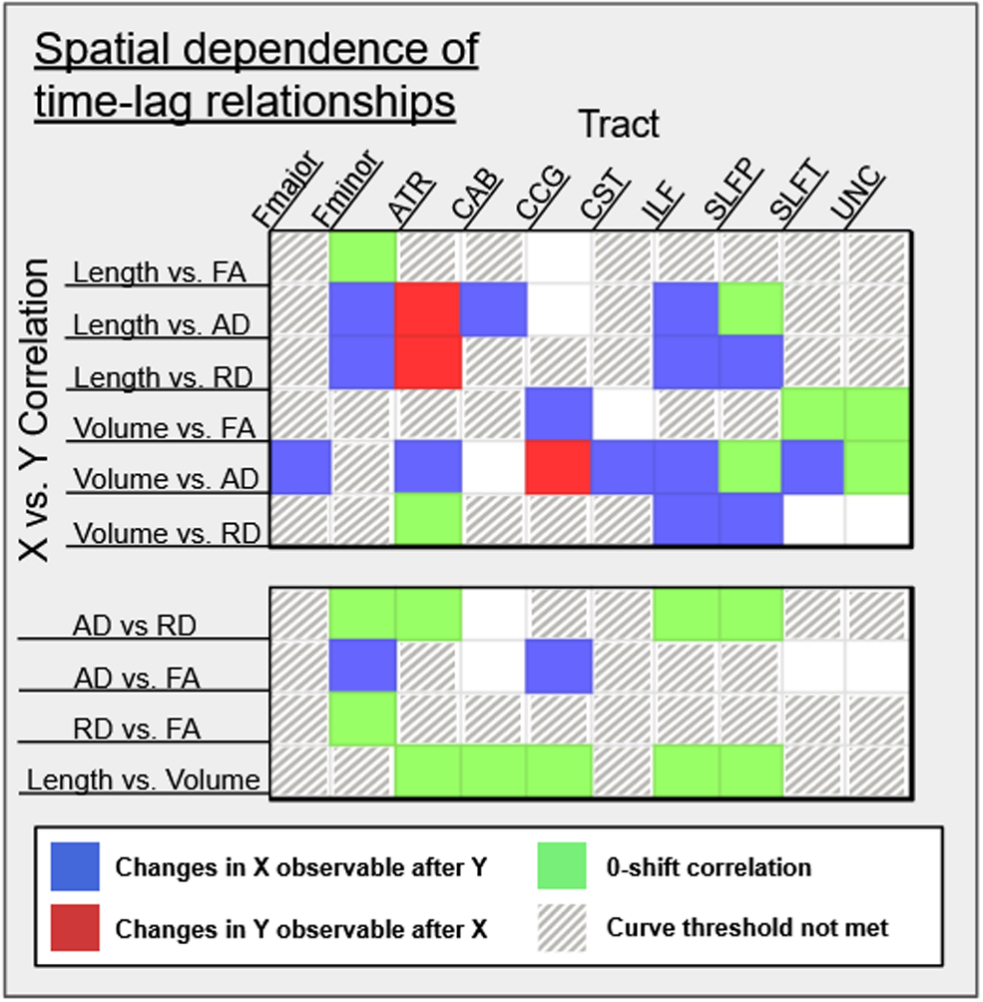
Tract-wise cross-correlations demonstrating significant peak correlations (*p*< .05) for pairs of morphometric and microstructural variables at positive, negative, and zero lag.

When correlating Length_perc_to Volume_perc_, we found significant zero-shift correlations in ATR, CAB, CCG, ILF, and SLFP. Significant zero-shift peak correlations between AD_perc_and RD_perc_were found in Fminor, ATR, ILF, and SLFP. Additionally, significant correlations between AD_perc_and FA_perc_were identified at negative correlational shift in Fminor and CCG such that changes in FA were detectable at younger ages than in AD_perc_, and between RD_perc_and FA_perc_at zero shift in the Fminor (Fig. 4).

### 3.4 Morphometry-microstructure relationships

#### 3.4.1 Whole-brain WM

Whole-WM FA, but not MD, was associated with morphometry independently of age and sex. Negative associations between age and FA_perc_, as well as positive associations between age and MD_perc_, were observed irrespective of sex and morphometric contributions. Sex differences were identified in whole-WM microstructure independent of age and morphometric variations, such that female subjects demonstrated higher mean MD_perc_across tracts (Table 2). A positive association between Length_perc_and FA_perc_and a negative association between VLR_perc_and FA_perc_were also observed across tracts independent of the effects of sex and age.

**Table 2.** Whole-WM relationships between age, sex, morphometry (length and VLR), and microstructure (FA and MD): list of regression coefficients.

	Percentage Length		Percentage VLR	
Tract	FA	MD	FA	MD
**All**	*F* (3, 496) = 11.31, *p* < .001, *R* ^2^_Adjusted_ = .06	*F* (3, 496) = 53.25, *p* < .001, *R* ^2^_Adjusted_ = .24	*F* (3, 496) = 13.02, *p* < .001, *R* ^2^_Adjusted_ = .07	*F* (3, 496) = 52.99, *p* < .001, *R* ^2^_Adjusted_ = .24
Morph.	0.22**	-0.035	-0.069***	-0.0033
Sex	0.0020	-0.0074*	0.0053	-0.0075*
Age	-0.00060**	0.0012***	-0.0011***	0.0013***

All models are multivariate linear regressions based on baseline-normalized measures (x_perc_) with overall F-statistics,*p*-values, and effect sizes (*R^2^_adjusted_*) listed for each section’s respective morphometry measure (“Morph.”), as well as sex and age. Slopes are noted below each regression equation and significant associations bolded with asterisks indicating significance. (*p< .05, **p< .01, ***p< .001), with positive coefficients indicating M > F for sex differences.

#### 3.4.2 Tract-wise WM

Examining tract-wise microstructure-Length_perc_associations (Table 3, Columns 2 and 3), Length_perc_was found to be positively associated with MD_perc_in ATR and negatively associated in CST irrespective of age and sex. Additionally, Length_perc_was positively associated with FA_perc_in Fminor, ATR, CAB, CST, ILF, SLFT, and negatively associated in UNC, when independent of age and sex. Examining tract-wise microstructure-VLR_perc_associations (Table 3, Columns 4 and 5), VLR_perc_was negatively associated with FA_perc_in Fmajor, Fminor, ATR, CAB, CCG, CST, ILF, and UNC, and positively associated in the SLFT, independent of age and sex. VLR_perc_was also negatively associated with MD_perc_in the Fmajor and positively associated in the CAB, again independent of age and sex effects.

**Table 3.** Tract-wise relationships between age, sex, morphometry (length and VLR), and microstructure (FA and MD): list of regression coefficients.

	Percentage Length		Percentage VLR	
Tract	FA	MD	FA	MD
**Fmajor**	*F* (3, 489) = 9.21, *p* < .001, *R* ^2^_Adjusted_ = .05	*F* (3, 489) = 30.00, *p* < .001, *R* ^2^_Adjusted_ = .15	*F* (3, 489) = 40.40, *p* < .001, *R* ^2^_Adjusted_ = .19	*F* (3, 489) = 32.90, *p* < .001, *R* ^2^_Adjusted_ = .16
Morph.	-0.058	0.056	-0.13***	-0.031**
Sex	-0.021***	-0.011**	0.0015	-0.0073
Age	-0.00056**	0.00099***	-0.0011***	0.00084***
**Fminor**	*F* (3, 489) = 26.61, *p* < .001, *R* ^2^_Adjusted_ = .14	*F* (3, 489) = 67.30, *p* < .001, *R* ^2^_Adjusted_ = .29	*F* (3, 489) = 38.7, *p* < .001, *R* ^2^_Adjusted_ = .19	*F* (3, 489) = 68.40, *p* < .001, *R* ^2^_Adjusted_ = .29
Morph.	0.14*	0.0059	-0.11***	-0.020
Sex	0.017**	-0.0062	0.0055	-0.0085
Age	-0.0014***	0.0020***	-0.0023***	0.0018***
**ATR**	*F* (3, 493) = 6.92, *p* <.001, *R* ^2^_Adjusted_ = .03	*F* (3, 493) = 67.80, *p* < .001, *R* ^2^_Adjusted_ = .19	*F* (3, 493) = 6.08, *p* < .001, *R* ^2^_Adjusted_ = .03	*F* (3, 493) = 64.60, *p* < .001, *R* ^2^_Adjusted_ = .28
Morph.	0.23**	0.11**	-0.060**	-0.014
Sex	0.0044	-0.0091*	0.0068	-0.0078
Age	-0.00044	0.0018***	-0.00070**	0.0017***
**CAB**	*F* (3, 486) = 11.53, *p* < .001, *R* ^2^_Adjusted_ = .06	*F* (3, 486) = 10.60, *p* < .001, *R* ^2^_Adjusted_ = .06	*F* (3, 486) = 49.40, *p* < .001, *R* ^2^_Adjusted_ = .23	*F* (3, 486) = 27.50, *p* < .001, *R* ^2^_Adjusted_ = .14
Morph.	0.20**	0.026	-0.22***	0.050***
Sex	-0.010	0.0025	0.0047	-0.00054
Age	-0.0013**	0.00074***	-0.0027***	0.00088***
**CCG**	*F* (3, 491) = 14.74, *p* < .001, *R* ^2^_Adjusted_ = .08	*F* (3, 491) = 39.30, *p* < .001, *R* ^2^_Adjusted_ = .19	*F* (3, 491) = 16.10, *p* < .001, *R* ^2^_Adjusted_ = .08	*F* (3, 491) = 39.20, *p* < .001, *R* ^2^_Adjusted_ = .19
Morph.	0.073	-0.028	-0.045	-0.0053
Sex	0.024*	-0.0044	0.022*	-0.0040
Age	-0.0018***	0.0013***	-0.0020***	0.0012***
**CST**	*F* (3, 487) = 11.20, *p* < .001, *R* ^2^_Adjusted_ = .06	*F* (3, 487) = 12.50, *p* < .001, *R* ^2^_Adjusted_ = .07	*F* (3, 487) = 7.11, *p* < .001, *R* ^2^_Adjusted_ = .04	*F* (3, 487) = 9.10, *p* < .001, *R* ^2^_Adjusted_ = .05
Morph.	0.33***	-0.15**	-0.087***	-0.0018
Sex	-0.0039	-0.014**	-0.0076	-0.015***
Age	0.000092	0.000438**	-0.00021	0.00052***
**ILF**	*F* (3, 493) = 17.60, *p* < .001, *R* ^2^_Adjusted_ = .09	*F* (3, 493) = 42.90, *p* < .001, *R* ^2^_Adjusted_ = .20	*F* (3, 493) = 16.50, *p* < .001, *R* ^2^_Adjusted_ = .09	*F* (3, 493) = 42.4, *p* < .001, *R* ^2^_Adjusted_ = .20
Morph.	0.25***	0.039	-0.066***	-0.00086
Sex	-0.014*	-0.014***	-0.011	-0.013**
Age	-0.00086***	0.0014***	-0.0013***	0.0014***
**SLFP**	*F* (3, 493) = 4.63, *p* < .001, *R* ^2^_Adjusted_ = .09	*F* (3, 493) = 47.30, *p* < .001, *R* ^2^_Adjusted_ = .22	*F* (3, 493) = 4.09, *p* = .006, *R* ^2^_Adjusted_ = .02	*F* (3, 493) = 4.09, *p* < .001, *R* ^2^_Adjusted_ = .22
Morph.	-0.083	-0.046	0.015	-0.011
Sex	0.012	-0.010*	0.012	-0.0092*
Age	-0.00071**	0.0014***	-0.00050*	0.0015***
**SLFT**	*F* (3, 491) = 3.65, *p* = .013, *R* ^2^_Adjusted_ = .02	*F* (3, 491) = 51.40, *p* < .001, *R* ^2^_Adjusted_ = .23	*F* (3, 491) = 3.74, *p* = .011, *R* ^2^_Adjusted_ = .02	*F* (3, 491) = 50.70, *p* < .001, *R* ^2^_Adjusted_ = .23
Morph.	0.11*	0.056	0.034*	-0.011
Sex	0.011	-0.0089*	0.0077	-0.0064
Age	-0.00010	0.0016***	-0.000075	0.0015***
**UNC**	*F* (3, 491) = 5.76, *p* < .001, *R* ^2^_Adjusted_ = .03	*F* (3, 491) = 45.30, *p* < .001, *R* ^2^_Adjusted_ = .21	*F* (3, 491) = 18.10, *p* < .001, *R* ^2^_Adjusted_ = .09	*F* (3, 491) = 44.80, *p* < .001, *R* ^2^_Adjusted_ = .21
Morph.	-0.10*	0.021	-0.14***	-0.0012
Sex	-0.015*	-0.0031	-0.010	-0.0025
Age	-0.00056*	0.0013***	-0.0011***	0.0013***

All models are multivariate linear regressions based on baseline-normalized measures (x_perc_) with overall F-statistics,*p*-values, and effect sizes (*R^2^_adjusted_*) listed for each section’s respective morphometry measure (“Morph.”), as well as sex and age. Slopes are noted below each regression equation and significant associations bolded with asterisks indicating significance. (*p< .05, **p< .01, ***p< .001), with positive coefficients indicating M > F for sex differences.

Negative associations between age and FA_perc_were also found in Fmajor, Fminor, CAB, CCG, ILF, SLFP, and UNC, with positive associations between age and MD_perc_in all tracts independent of sex and Length_perc_. Negative age associations with FA_perc_were found in Fmajor, Fminor, ATR, CAB, CCG, ILF, SLFP, and UNC, while positive age associations with MD_perc_were identified in all tracts irrespective of sex and VLR_perc_.

Regarding sex differences, significant differences in MD_perc_were identified in Fmajor, ATR, CST, ILF, SLFP, and SLFT, independent of age or Length_perc_, with higher values in female subjects in all cases. Significant sex-related differences in FA_perc_(being higher in females) were found in Fmajor, ILF, and UNC independent of age and Length_perc_. By the same token, FA_perc_in male subjects was higher in Fminor, CCG. Significant differences in MD_perc_irrespective of VLR_perc_and age indicate that female subjects demonstrated higher MD_perc_in CST, ILF, and SLFP. Similarly, significant differences were observed in FA_perc_in the CCG alone (with higher values in male subjects) independent of VLR_perc_and age effects.

To summarize,Table 2also imparts some general trends segregating different tracts. The dominant trend is for tracts to demonstrate significant associations linking microstructure with all dependent variables, including morphometry, sex, and age, with a few notable exceptions. Fmajor, Fminor, ATR, SLFT, and UNC deviate from the general trend in that tract microstructure differed by sex independently of its associations with Length_perc_and age; this was not the case in the comparable Length_perc_-VLR_perc_, analysis, in which microstructure did not differ by sex. Moreover, CAB demonstrated no effect by sex in either analysis. Additionally, in the Fmajor, microstructure was significantly associated with VLR_perc_, but not with Length_perc_. No relationships between morphometry and microstructure were found in CCG and SLFP, independent of associations between microstructure and both sex and age.

### 3.5 Tract-wise canonical correlations

**Fig. 5.**
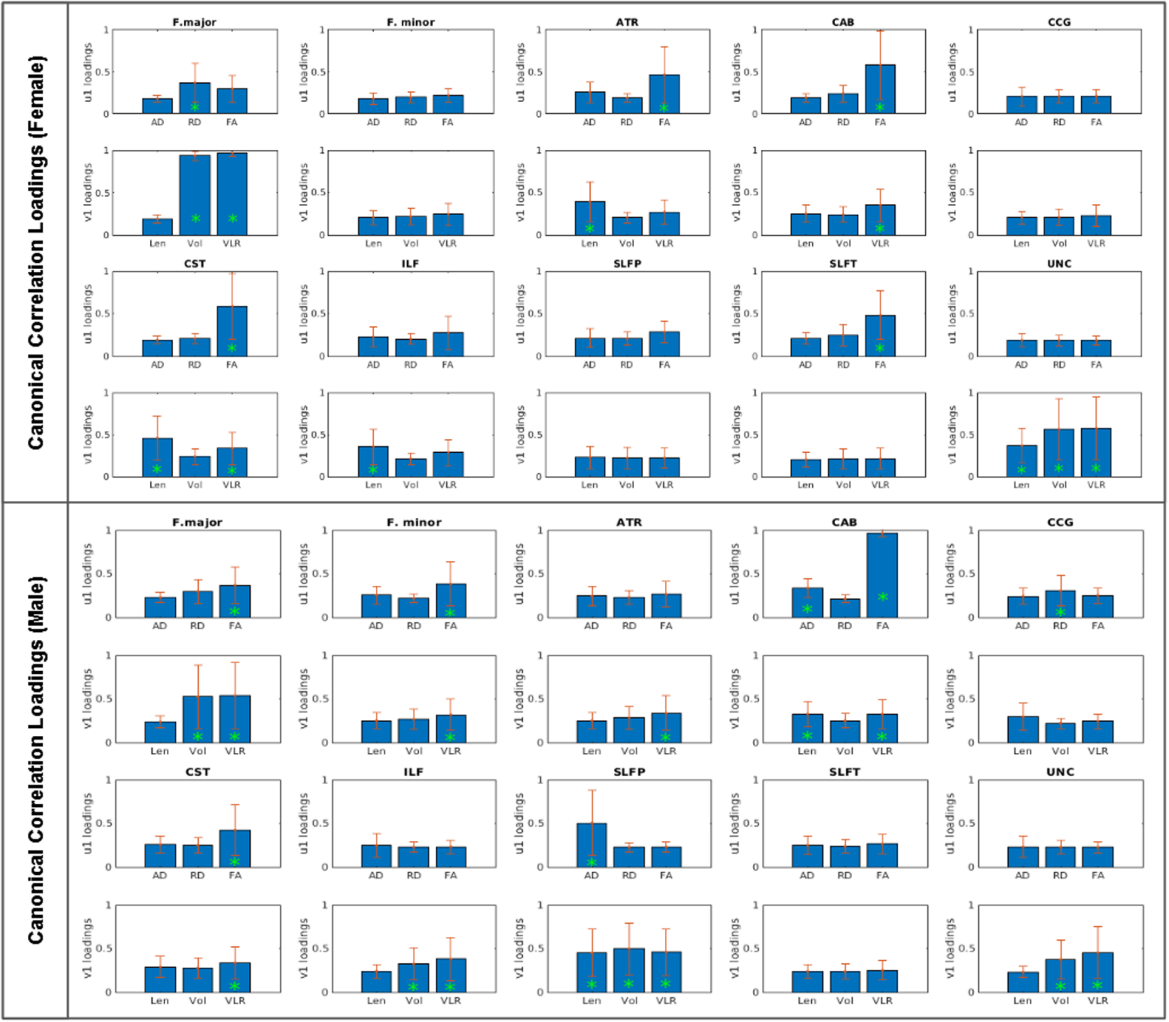
Tract-wise contributions by all microstructural (AD_perc_, RD_perc_, FA_perc_) and morphometric (Length_perc_, Volume_perc_, VLR_perc_) measures to categorical microstructural-morphometric relationships in aging. U1 and V1 refer to latent variables related to microstructure and morphometry, respectively. Green asterisks denote the loading threshold of 0.3.

Given the sex differences observed in multivariate regression, CCA was performed on the sexes separately in addition to the full-sample analysis. CCA identified varying patterns of tract-wise joint microstructural and morphometric loadings between male and female subjects (Fig. 5andSupplementary Table 4). Loadings are listed only for statistically significant (*p*< .05) canonical correlations.

**Fig. 6.**
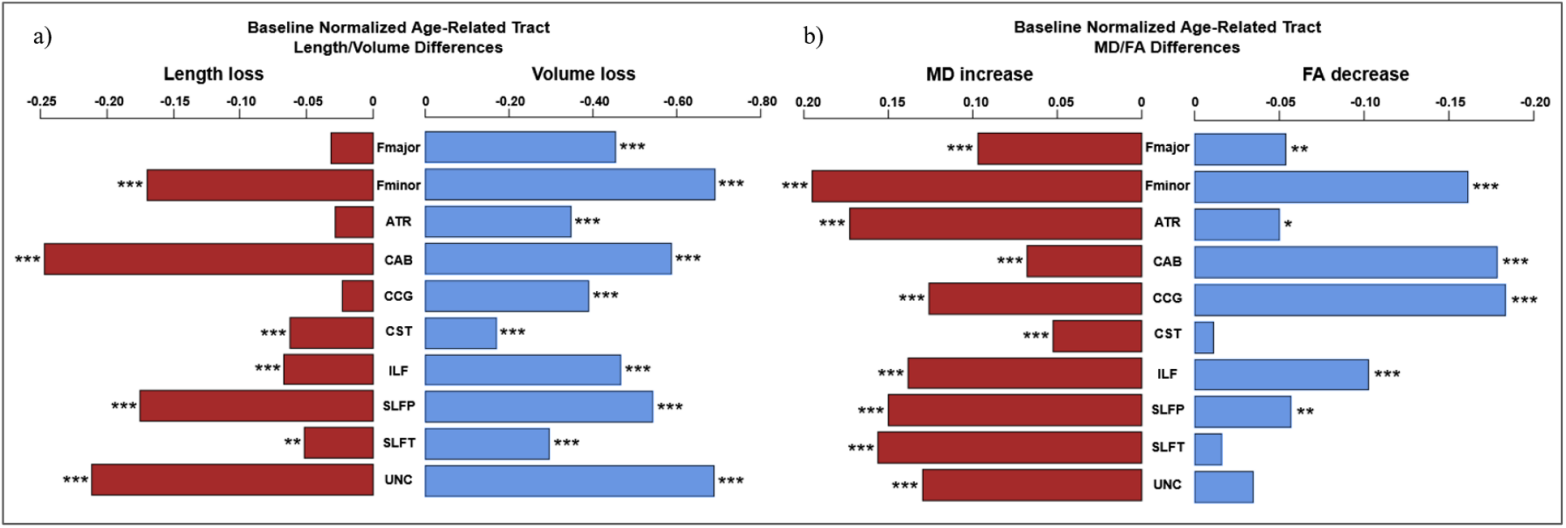
Summary comparison of tract-wise age effects on morphometric and microstructural variables. (a) A comparison of age effects on Length_perc_and Volume_perc_, (b) A comparison of age effects on MD_perc_and FA_perc_. Significant effects of age are denoted with asterisks (***p< .001, **p< .01, *p< .05).

In female subjects, strong loadings (>0.3) relating microstructure and morphometry were identified in Fmajor, ATR, CAB, and CST. FA_perc_demonstrated the most common significant microstructural loading across three tracts (ATR, CAB, CST), while RD_perc_loading contributed significantly to the microstructural-morphometric correlations (canonical correlations) in the Fmajor alone. Meanwhile, Length_perc_was a substantial morphometric contributor to the canonical correlation in female subjects in ATR and CST, Volume_perc_was a substantial contributor to the canonical correlation in Fmajor alone, and VLR_perc_in Fmajor, CAB, and CST. In cases where only one category constituted a substantial contributor, the ILF’s canonical correlation was driven mainly by Length, while the SLFT’s was driven mainly by FA, and UNC’s was driven by all morphometric but no microstructural variables.

Male subjects demonstrated strong loadings linking microstructure and morphometry in the Fmajor, Fminor, CAB, CST, and SLFP. FA_perc_was again found to be the most commonly microstructural loading contributor to the canonical correlation, with additional loading contributions by AD_perc_in the CAB, and AD_perc_loading in the absence of FA_perc_loading in the SLFP. With regards to morphometry, Length was a substantial morphometric contributor to the canonical correlation in male subjects in the CAB and SLFP, while Volume was a substantial contributor to the canonical correlation in Fmajor and SLFP. VLR was a substantial contributor to the canonical correlation in Fmajor, Fminor, CAB, CST, and SLFP. Notably, both the ILF and UNC’s canonical correlations have overwhelmingly morphometric drivers.

## 4 Discussion

Our initial hypotheses stated that (1) age-related differences in microstructure would be detectable earlier in the lifespan than those in morphometry, (2) age-related microstructural differences would be greater in tracts that demonstrate greater age-related morphometrical differences, (3) relationships between age-related differences in morphometry and microstructure would differ by sex, and (4) there would be interactions between age-related differences in fiber length and volume, with tracts that become thinner with age also having higher mean tract lengths. Our findings generally support hypotheses (1), (2), and (3), but no strong support for hypothesis (4). In the following, we discuss these findings in more detail.

### 4.1 Morphometry/microstructural differences in age-associated correlations

In addressing hypothesis (1), results from the CCF analysis demonstrated that microstructural changes were detectable at a younger age than morphometric changes in the majority of tracts. Given the relatively linear declines observed in FA measures across analyses, the majority of observations derived from CCF involve the more nonlinear AD and RD age effects, both of which lead those in morphometric variables (indicated in blue inFig. 4). One interpretation for this finding is that WM microstructural variations precede those detectable in morphometry, as suggested by histology (Hugenschmidt et al., 2008). Of course, this could also stem from inherent sensitivities in the microstructural and morphometric parameters chosen in this study. These findings remain exploratory, as a longitudinal design would be needed to ascertain temporal precedence in different age effects. Notable exceptions were the ATR, in which length changes appear to be detectable at earlier ages than diffusion measures, as well as the CST, which showed significant correlations at both negative and positive correlational shifts depending on measure. While these run counter to the intuitive assumption that makes up hypothesis (1), the differences in measurement sensitivities of both microstructural and morphometric approaches may further complicate the interpretation.

### 4.2 Morphometry and microstructural associations across ages

Contrary to hypothesis (2), we found that while consistent trends in baseline-normalized morphometric and microstructural declines with age were identified in the majority of tracts, there was no single unifying trend in the relationship between morphometry and microstructure, as shown inTable 3. However, the majority of tracts demonstrate microstructure-morphometry associations even when controlling for the effects of age on microstructural declines, lending support to hypothesis (2); specifically, higher FA and lower MD tends to be associated with higher morphometric variables (Fig. 6). This suggests that the microstructural-morphometric relationships, while present, may be complicated by other differentiating factors in WM aging. Notably, stronger microstructural-morphometric associations were observed in more anterior and longitudinal tracts, which may suggest limited support for regional differences similar to those proposed by retrogenesis theories (Raz, 2000;Robinson et al., 2024;Slater et al., 2019). Note that while normalizing morphometric measures by anatomic variables such as intracranial volume (ICV) was incorporated in early versions of this study, the need to make comparisons between morphometry and baseline-normalized microstructural measures and a lack of an “ICV equivalent” by which to normalize tract length resulted in our extending baseline normalization across all measures.

#### 4.2.1 Tract-wise microstructure and age

Relationships between age and WM integrity across tracts reflect the expected trends of increasing MD_perc_and decreasing FA_perc_(fractional MD and FA relative to the young baseline) with advancing age. This echoes general findings in the literature regarding microstructural WM decline (Benitez et al., 2018;Sala et al., 2012). As in the morphometric-age associations, tracts varied considerably in their trajectory of microstructural changes. While all tracts demonstrated increases in MD_perc_, three tracts, namely the CST, SLFT, and UNC, exhibited no significant effects of age on FA_perc_. This is puzzling given the common observation of robust MD-FA anti-correlations. A possible explanation for this observation is the contribution of crossing fibers to diffusion measures within a tract, such as regional increases in FA due to degeneration of crossing fibers, despite relatively maintained MD. Moreover, increasing AD can potentially be due to the deterioration of crossing fibers resulting in RD increase of the secondary (perpendicular) fiber direction, which translates to AD increases in the principal fiber direction, with maintained MD (Chad et al., 2018;Han et al., 2023). Although the interpretation of increasing FA as selective degeneration of crossing fibers in aging is relatively recent, its validity was recently demonstrated in cross-sectional data (Chad et al., 2018;Han et al., 2023). As both the SLFT and UNC are relatively posterior tracts, these findings may be interpretable as support for regionally-driven retrogenesis theories on brain aging (i.e., an anterior-posterior gradient) (Brickman et al., 2012;Hoagey et al., 2019;Madden et al., 2012;Slater et al., 2019;Sullivan et al., 2006). Despite the weak FA-age associations in these tracts, significant MD_perc_increases with age were identified in all, suggesting ongoing (albeit weaker than in anterior tracts) age-related degeneration. To further clarify the implied signatures of WM aging, these cross-sectional findings also need to be compared with longitudinal data.

#### 4.2.2 Tract-wise morphometry and age

In line with existing research on white matter atrophy (de Groot et al., 2015;Sala et al., 2012;Tang et al., 1997), we observed broad age-related decreases in all three raw morphometric measures, with annual variations in VLR_perc_surpassing that of Length_perc_. This indicates that in normal aging, tracts thin more than they shorten, proportionally speaking. As VLR represents the cross-sectional area, even if all area dimensions decreased with age at a similar rate, VLR would still appear to decline more rapidly than Length. We also note considerable heterogeneity in macrostructural age changes across the WM in spite of more homogenous rates of microstructural deterioration, similar to previous work (Schilling et al., 2022), demonstrating the need to examine relationships between morphometry and microstructure to further clarify the basis of tract-wise differences.

At the tract level, decreasing raw tract volume in aging was found in all tested tracts except in the CST. Conversely, the Fmajor and Fminor exhibited notably greater age-related raw volume differences than other WM regions, suggesting excessive shrinkage in these two regions. Moreover, similar to findings of microstructure, the CST shows considerable robustness to morphometric declines. Furthermore, the UNC showed strong age effects on morphometry in both Length_perc_and Volume_perc_, aligning with observations of increasing MD_perc_described earlier. These findings corroborate existing research on tract-wise differences in microstructural aging, as well as recent work noting broad morphometric declines across the brain, with commissural tracts as pathways with relatively high susceptibility to age-related change and sensorimotor tracts such as the CST relatively preserved (de Groot et al., 2015;Schilling et al., 2022). Notably, the Fmajor, ATR, and CCG do not significantly shorten in aging (Fig. 2). A speculative cause for these tract-wise differences in age effects includes the retrogenesis hypotheses that later-developing anterior commissural tracts are more susceptible to age-related declines (de Groot et al., 2015), but the morphometric declines in our data do not seem to follow a clear anterior-posterior gradient.

Tract-wise regression of morphometry against microstructure showed a consistent presence of microstructure-morphometry associations across the WM, in support of hypothesis (2). However, even while controlling for the impacts of both age and sex, the associations varied enough to preclude the presence of a single shared trend. For instance, three of 10 tracts (Fmajor, CCG, SLFP) showed no such association in at least one morphometric measure. Thus, while the relationship exists as posted in hypothesis (2), it is also more complex than we initially expected.

### 4.3 Sex differences in morphometry and microstructure

The conversion of microstructural metrics to percent-of-the-baseline values appeared to have enhanced the sex differences in these metrics, in support of hypothesis (3). Following baseline normalization, while males demonstrated higher values across measures in a majority of tracts, exceptions were identified in several tracts (Fmajor, Fminor, CCG, and CST) in which females showed significantly higher values in at least one morphometric measure that were not observable prior to normalization. These differences also extend to microstructure. Specifically, females exhibited higher fractional MD (MD_perc_) increase than males, implying more rapid microstructural degeneration in female aging, while directionality of FA_perc_differences was more varied. Evidence of sex differences in aging trajectories was reinforced by significant differences in age effects between sexes identified in several measures, most notably in CAB, SLFP, and SLFT (Supplementary Table 2;Supplementary Fig. 4). These differences are at the heart of our motivation to study these relationships in order to better understand the WM aging process as is visible through different imaging modalities. Furthermore, these findings demonstrated the importance of examining relationships between and within microstructural and morphometric categories of measures while examining sex differences.

### 4.4 Understanding patterns of morphometric-microstructural aging through multimodal integration

The fusion of morphometric and microstructural observations in CCA allowed us to examine regional differences in integrated patterns of WM aging in view of expected sex differences. The CCA provided a succinct way to summarize the variation in age-related microstructural-morphometric associations. Variables with strong loadings are considered main drivers of the significant canonical correlation between morphometry and microstructure.

The predominance of tracts demonstrating significant FA loading in the relationship between microstructure and morphometry suggests that changing anisotropy, theoretically driven by the breakdown of axon fibers and resulting shifts in relative diffusion gradients (Marner et al., 2003;Tang et al., 1997), displays the strongest association with morphometric changes in aging. However, the presence of tracts exhibiting significant loading by AD or RD, both in addition to and in the absence of FA loading, suggests a variety of signatures or stages of microstructural decline present across tracts. In addition, the variety of morphometric loadings suggests multiple patterns of morphometric decline rather than uniform atrophy (i.e., microstructural variations being related to thinning vs. shortening). However, the presence of sex differences in these patterns suggests the modes of decline in different tracts may not be shared across sexes, an interesting question requiring dedicated investigation. Of course, it is also possible that tract-wise differences in patterns of morphometric change are driven by other anatomical variables, such as regional differences in axon diameter or the availability and resilience of vascular sources (Bernier et al., 2020;Chen et al., 2011;Fan et al., 2020;Huang et al., 2020;Liewald et al., 2014), a topic that is also worthy of dedicated investigation (Robinson et al., 2024).

Overall, female subjects appear more likely to demonstrate significant Length loading in microstructural-morphometric associations (in 3/4 of the tracts with significant canonical correlations), while male subjects showed significant Length loading in only 2 out of 5. In other words, morphometric-microstructural correlations are more likely to be driven by shortening tractss in females than males. VLR (i.e., cross-sectional area) loading appears to be the most common morphometric contribution across sexes. Only in the female ATR did we identify fiber shortening, not thinning, driving the canonical correlations.

Taken together, these findings suggest fiber length and FA covary in aging more than any other pair of metrics in females, signifying potentially different regimes of WM degeneration between the sexes. A driving source of this sex discrepancy may also be the hormonal effects of menopause in female subjects, as the impact of menopause on the brain has been demonstrated to include structural, connectivity, and metabolic effects (Mosconi et al., 2021). Our results likely capture structural effects of peri-menopause and post-menopause on the aging WM. A promising future direction is to relate these differences to fundamental physiological attributes such as fiber caliber.

### 4.5 Existence of culling and survival bias

Contrary to hypothesis (4), our results do not show a dominant trend of tract volume being anti-correlated with mean tract length. Tracts such as the CST shorten with age with relatively little proportionate losses in volume. In contrast, tracts such as the CCG, ATR, and Fmajor lose Volume_perc_with relatively little loss in tract Length_perc_. We suggest that we may be observing different tracts at different stages of decline with varying levels of packing density (Fan et al., 2020;Huang et al., 2020). Specifically, reduction in mean tract length with relatively preserved volume may be the result of reduced packing density while losing fibers. This could be a result of increased size of surviving fibers as well as thickening of myelin in aging axons, resulting in notably larger mean diameter in the aging tract (Tang et al., 1997). The geometry of packing larger fibers produces an increase in extracellular space between the fibers and a relatively lower impact on tract volume. This is in contrast to an even degeneration of both large and small fibers, which could allow a more densely packed bundle and produces relatively greater losses in tract volume, as we have seen in Fmajor, ATR, and CCG (Marner et al., 2003;Tang et al., 1997).

Sex differences in these three tracts were also identified in regression analysis independent of the effects of age and Length_perc_, but not VLR_perc_. It is possible that these differences across sexes and tracts may be due to the known regional and sex-related variations in vascularization and perfusion decline in aging (Chen et al., 2011), which is the focus of our other ongoing work (Robinson et al., 2023).

### 4.6 Limitations

While the intent of this study was to lay the initial groundwork for multimodal integration for understanding white matter aging, the choice to apply widely applied metrics such as MD and FA introduces a number of limitations that may be resolved in more advanced diffusion modeling. While hypertension, a leading vascular-risk factor, is typical in a healthy aging population, we do not investigate the role of vascular risk in our study, as this would be beyond the scope of the current research question. Moreover, the preponderance of weak or absent FA decline with advancing age suggests the importance of recognizing the role of crossing fibers in DTI studies of WM aging. Although we have recently provided substantial experimental evidence to support the existence of selective degeneration of crossing fibers (Chad et al., 2018;Han et al., 2023), our application of this interpretation in this study remains to be directly tested. Moreover, this study chose to focus on only the 10 major WM tracts. While this provides lower spatial WM coverage than more recent segmentations (Maffei et al., 2021), the choice was made to both minimize the degree of overlap/partial-voluming between tract segmentations, as well as to aid in comparisons with previous research utilizing TRACULA. Additionally, as the exploratory CCF analysis used to highlight differences in microstructure-morphometry correlations across age groups depends on nonlinearity in the age associations of its inputs, the heterogeneity in the presence of significant quadratic versus linear age effects observed across measures restricted the tracts that can be submitted to cross-measure tract-wise comparisons. Finally, while our methods are novel, the cross-sectional nature of the HCP-A dataset merits future validations of our findings using a longitudinal dataset. Thus, our future work will build on these findings to explore both longitudinal covariations in microstructure and morphometry, their association with cortical age-related variations, and differences across hemispheres in each sex.

## 5 Conclusions

Both morphometry and microstructure analyzed in this study depict WM age-related differences as highly tract-specific and sex-specific. Tract-wise degeneration of microstructure was found to be detectable at younger ages than morphometry across the majority of tracts, suggesting that microstructural deterioration may occur prior to morphometric changes. Moreover, the two types of variations are significantly correlated in the majority of tracts. Different microstructural attributes are associated with different aspects of tract morphometry across tracts, with the anterior and longitudinal tracts demonstrating the strongest microstructural-morphometry associations. Extending these comparisons across longitudinal analysis will likely clarify relationships that cannot be readily observed cross-sectionally. Furthermore, while our current results do not illustrate a single shared microstructural-morphometric association across tracts, they do not preclude the possibility that additional variables, such as WM perfusion (Chen et al., 2013;Kim et al., 2020), are needed in achieving a more spatially consistent understanding of WM aging.

## Supplementary Material

Supplementary Material

## Data Availability

Data analyzed in this study were drawn from the Human Connectome Project in Aging (HCP-A) (Bookheimer et al., 2019). All included HCP-A datasets were made available under appropriate data usage agreements and can be accessed via the NIMH Data Archive (https://nda.nih.gov/general-query.%20html?q=%C2%A0query%3D%C2%A0featured-datasets:Human%20Connectome%20Projects%20(HCP)). Original code written in Matlab and R for data processing and analysis can be made available on request.
