# Supplementary material for: Uncovering patterns of white matter degeneration in normal aging: Links between morphometry and microstructure
Source: Imaging Neurosci (Camb). 2024 Aug 2;2:imag-2-00247. doi: 10.1162/imag_a_00247 (PMC12272242; doi:10.1162/imag_a_00247)
Supplement: Supplementary Material [file imag_a_00247-supp.pdf]

Supplementary Materials

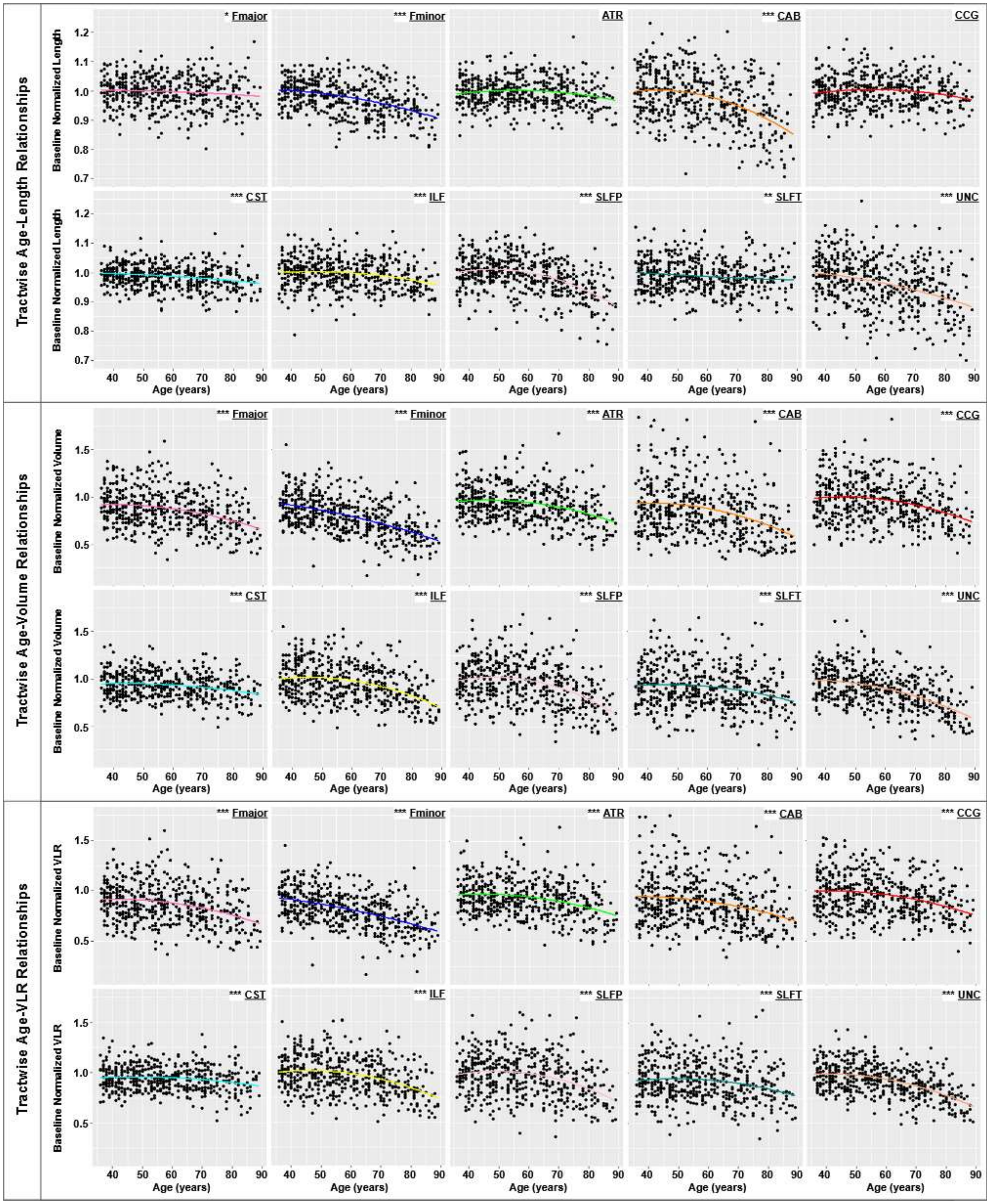

Supplementary Figure 1*: Tractwise regression of $Length_{perc}$, and $Volume_{perc}$, and VLR_{perc} against age with each dot representing one subject. Significant quadratic effects of age are denoted with asterisks (***: p<.001, **: p<.01, *: p<.05)*

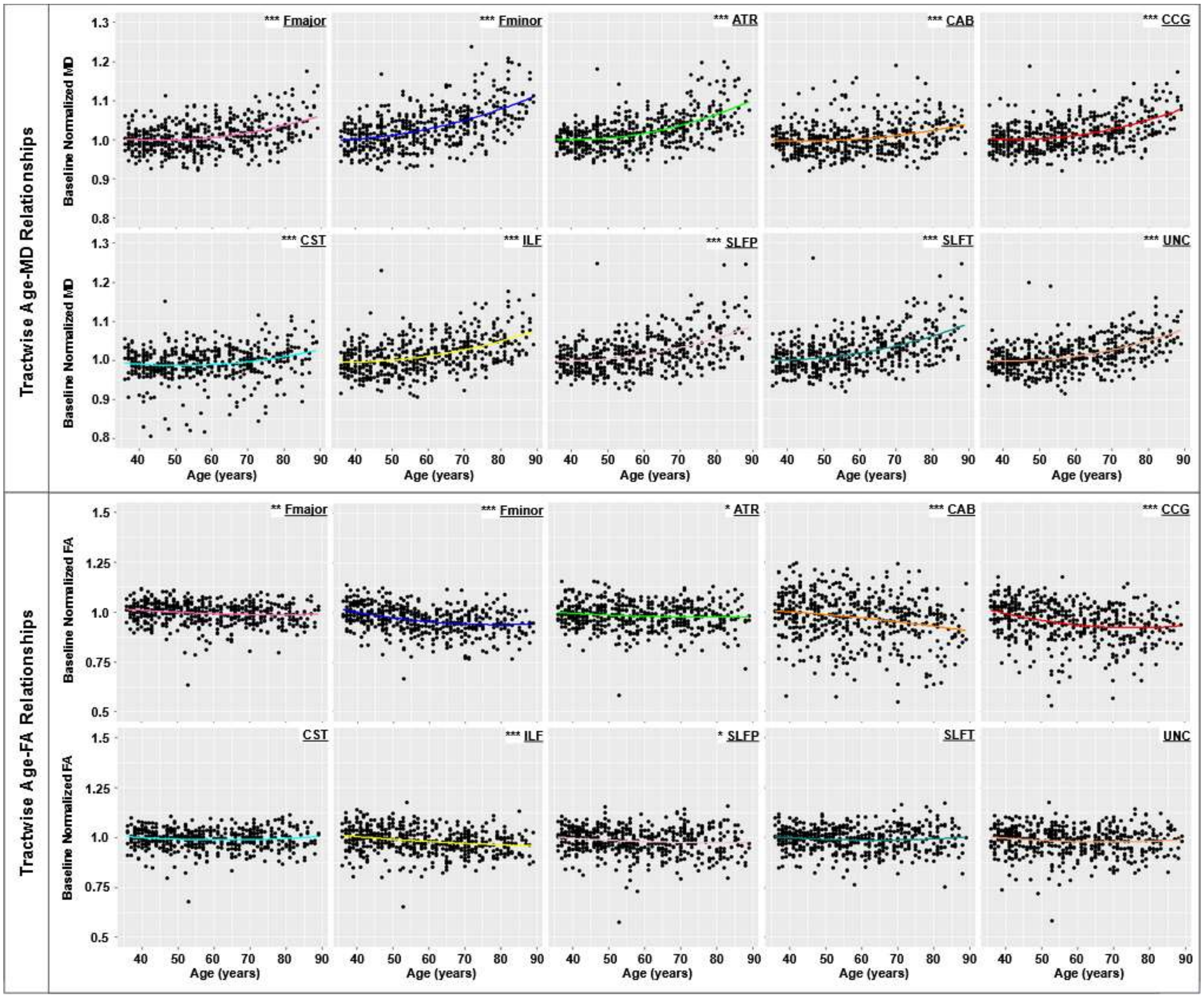

Supplementary Figure 2: *Tractwise quadratic regression of age effects on MD_{perc} and FA_{perc} with each dot representing one subject. Significant quadratic effects of age are denoted with asterisks (***: p<.001, **: p<.01, *: p<.05)*

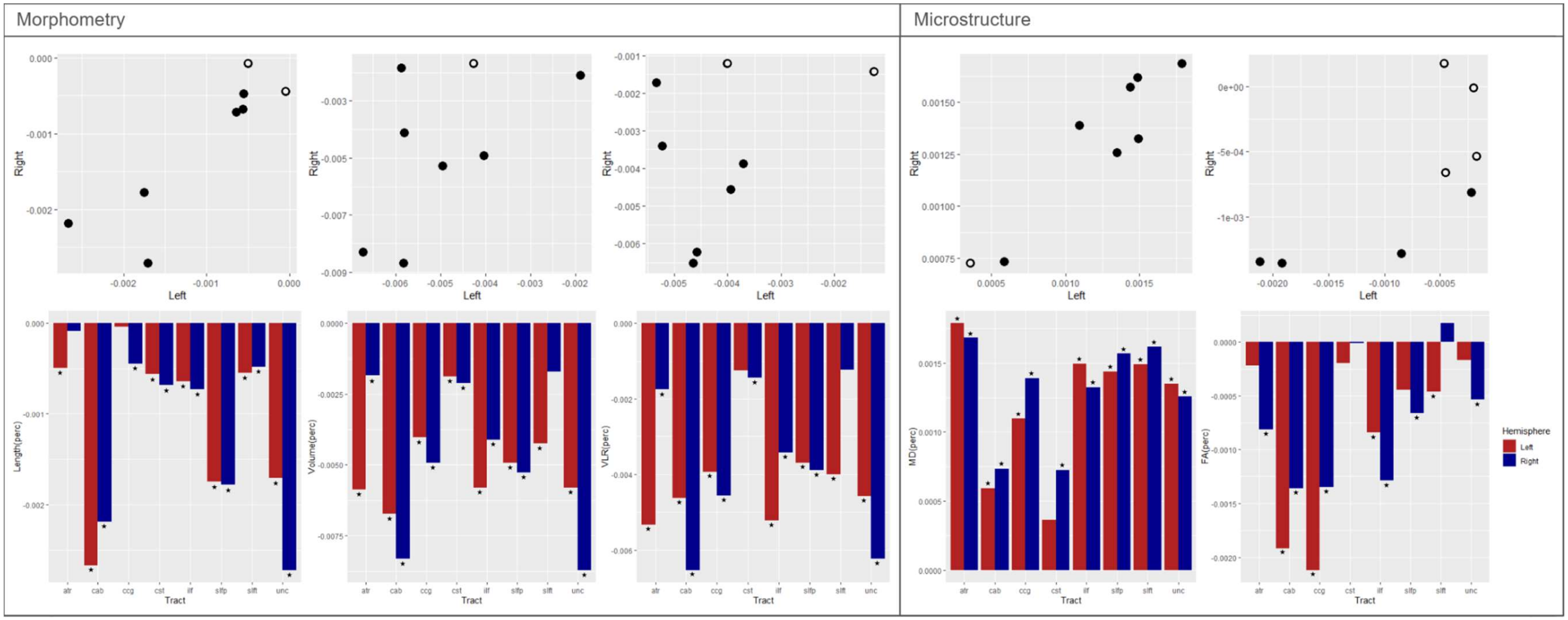

Supplementary Figure 3: *Tractwise regression of age effects on Length$_{perc}$, Volume$_{perc}$, VLR$_{perc}$, MD$_{perc}$, and FA$_{perc}$ divided by hemisphere with scatterplots demonstrating tracts which do (dot) and do not (circle) share significance between hemispheres. Significant linear effects of age are denoted with asterisks (***: p<.001, **: p<.01, *: p<.05)*

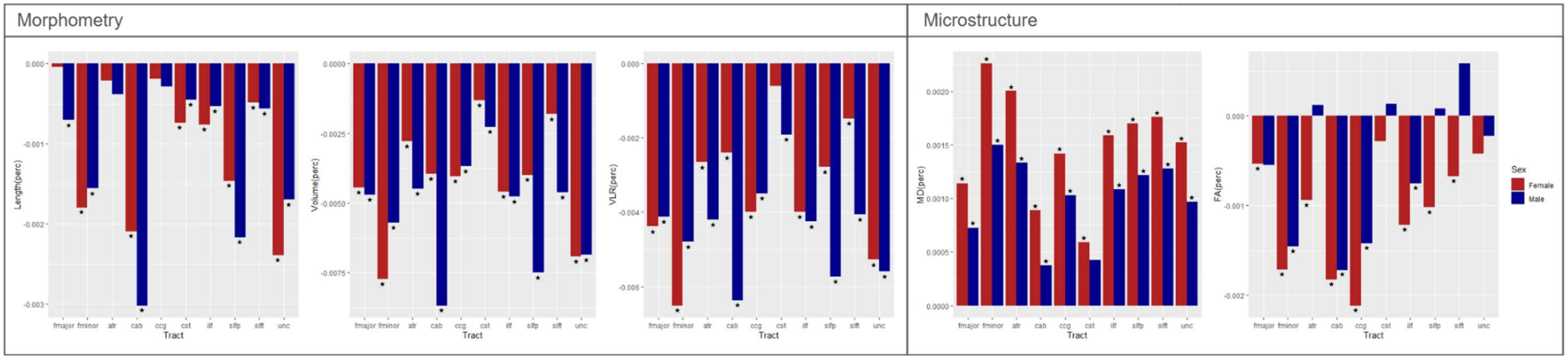

Supplementary Figure 4: *Tractwise regression of age effects on Length$_{perc}$, Volume$_{perc}$, VLR$_{perc}$, MD$_{perc}$, and FA$_{perc}$ divided by sex. Significant linear effects of age are denoted with asterisks (***: p<.001, **: p<.01, *: p<.05)*

Supplemental Table 1: Tractwise age effects and sex differences on morphometry (length, Volume, and VLR) and microstructure (FA and MD): list of regression coefficients.
All models are multivariate linear regressions based on baseline-normalized measures (x_{perc}) with overall F-statistics, *p*-values, and effect sizes ($R^2_{adjusted}$) listed. Slopes are noted below each regression equation and significant associations bolded with asterisks indicating significance. (*:p<.05, **:p<.01, ***:p<.001), with positive coefficients indicating M>F for sex differences.

	**Morphometry**			**Microstructure**	
Tract	$Length_{perc}$	$Volume_{perc}$	VLR_{perc}	FA_{perc}	MD_{perc}
**Fmajor**	$F(2, 490) = 10.7$, $p < .001$, $R^2_{Adjusted} = .04$	$F(2, 490) = 64.2$, $p < .001$, $R^2_{Adjusted} = .20$	$F(2, 490) = 82.4$, $p < .001$, $R^2_{Adjusted} = .25$	$F(2, 490) = 13.0$, $p < .001$, $R^2_{Adjusted} = .05$	$F(2, 490) = 42.9$, $p < .001$, $R^2_{Adjusted} = .15$
Age	-0.000313	**-0.004547*** **	**-0.00427*** **	**-0.000539** **	**0.000968*** **
Sex	**-0.020543*** **	**0.141029*** **	**0.16519*** **	**-0.019620*** **	**-0.012479*** **
**Fminor**	$F(2, 490) = 51.6$, $p < .001$, $R^2_{Adjusted} = .17$	$F(2, 490) = 120$, $p < .001$, $R^2_{Adjusted} = .33$	$F(2, 490) = 114$, $p < .001$, $R^2_{Adjusted} = .32$	$F(2, 490) = 36.2$, $p < .001$, $R^2_{Adjusted} = .13$	$F(2, 490) =101$ $p < .001$, $R^2_{Adjusted} = .29$
Age	**-0.001698*** **	**-0.006910*** **	**-0.005816**	**-0.00161*** **	**0.001952*** **
Sex	0.007233	**-0.006910*** **	**-0.113368**	**0.01841** **	-0.006178
**ATR**	$F(2, 494) = 6.64$, $p = .001$, $R^2_{Adjusted} = .02$	$F(2, 494) = 18.7$, $p < .001$, $R^2_{Adjusted} = .07$	$F(2, 494) = 20.1$, $p < .001$, $R^2_{Adjusted} = .07$	$F(2, 494) = 3.44$, $p = .033$, $R^2_{Adjusted} = .01$	$F(2, 494) = 95.8$, $p < .001$, $R^2_{Adjusted} = .28$
Age	-0.000283	**-0.003478*** **	**-0.003282*** **	**-0.000501***	**0.001731*** **
Sex	**0.014326** **	0.000579	-0.014658	0.007642	**-0.007571***
**CAB**	$F(2, 487) = 41.9$, $p < .001$, $R^2_{Adjusted} = .14$	$F(2, 487) = 24.1$, $p < .001$, $R^2_{Adjusted} = .09$	$F(2, 487) = 17.2$, $p < .001$, $R^2_{Adjusted} = .06$	$F(2, 487) = 12.0$, $p < .001$, $R^2_{Adjusted} = .04$	$F(2, 487) = 15.1$, $p < .001$, $R^2_{Adjusted} = .05$
Age	**-0.00247*** **	**-0.00588*** **	**-0.004014*** **	**-0.001787**	**0.000680*** **
Sex	0.00527	**0.06433***	**0.063127** **	-0.009033	0.002621
**CCG**	$F(2, 492) = 5.92$, $p = .003$, $R^2_{Adjusted} = .02$	$F(2, 492) = 17.9$, $p < .001$, $R^2_{Adjusted} = .06$	$F(2, 492) = 18.0$, $p < .001$, $R^2_{Adjusted} = .06$	$F(2, 492) = 21.7$, $p < .001$, $R^2_{Adjusted} = .08$	$F(2, 492) = 58.6$, $p < .001$, $R^2_{Adjusted} = .19$
Age	-0.000227	**-0.003905*** **	**-0.003793*** **	**-0.001838*** **	**0.001261*** **
Sex	**-0.015412** **	-0.031338	-0.015771	**0.023190** **	-0.003945
**CST**	$F(2, 488) = 13.4$, $p < .001$, $R^2_{Adjusted} = .05$	$F(2, 488) = 23.4$, $p < .001$, $R^2_{Adjusted} = .08$	$F(2, 488) = 22.3$, $p < .001$, $R^2_{Adjusted} = .08$	$F(2, 488) = 0.35$, $p = .704$, $R^2_{Adjusted} = -.003$	$F(2, 488) = 13.7$, $p < .001$, $R^2_{Adjusted} = .05$
Age	**-0.000622*** **	**-0.001698*** **	**-0.001139** **	-0.000113	**0.000522*** **
Sex	0.005688	**-0.060709*** **	**-0.063444*** **	-0.002064	**-0.014733*** **

**ILF**	$F(2, 494) = 11.9$, $p < .001$, $R^2_{Adjusted} = .04$	$F(2, 494) = 32.6$, $p < .001$, $R^2_{Adjusted} = .11$	$F(2, 494) = 28.8$, $p < .001$, $R^2_{Adjusted} = .10$	$F(2, 494) = 15.9$, $p < .001$, $R^2_{Adjusted} = .06$	$F(2, 494) = 63.7$, $p < .001$, $R^2_{Adjusted} = .20$
Age	**-0.000668*** **	**-0.0046687*** **	**-0.004098*** **	**-0.001027*** **	**0.001384*** **
Sex	**0.011919** **	-0.0000645	-0.009869	-0.010760	**-0.013093*** **
**SLFP**	$F(2, 494) = 46.4$, $p < .001$, $R^2_{Adjusted} = .16$	$F(2, 494) = 32.4$, $p < .001$, $R^2_{Adjusted} = .11$	$F(2, 494) = 21.5$, $p < .001$, $R^2_{Adjusted} = .08$	$F(2, 494) = 5.58$, $p = .004$, $R^2_{Adjusted} = .02$	$F(2, 494) = 69.6$, $p < .001$, $R^2_{Adjusted} = .22$
Age	**-0.001753*** **	**-0.005432*** **	**-0.003991*** **	**-0.000566** **	**0.001503*** **
Sex	-0.007213	**0.040143***	**0.043030***	**0.012778***	**-0.009638***
**SLFT**	$F(2, 492) = 7.73$, $p < .001$, $R^2_{Adjusted} = .03$	$F(2, 492) = 51.9$, $p < .001$, $R^2_{Adjusted} = .17$	$F(2, 492) = 51.5$, $p < .001$, $R^2_{Adjusted} = .17$	$F(2, 492) = 3.0$, $p = .051$, $R^2_{Adjusted} = .008$	$F(2, 492) = 75.5$, $p < .001$, $R^2_{Adjusted} = .23$
Age	**-0.000519** **	**-0.002956*** **	**-0.002530*** **	-0.000160	**0.001564*** **
Sex	**0.013369** **	**0.170106*** **	**0.156502*** **	**0.012958***	**-0.008168***
**UNC**	$F(2, 492) = 40.1$, $p < .001$, $R^2_{Adjusted} = .14$	$F(2, 492) = 86.6$, $p < .001$, $R^2_{Adjusted} = .26$	$F(2, 492) = 81.5$, $p < .001$, $R^2_{Adjusted} = .25$	$F(2, 492) = 5.06$, $p = .007$, $R^2_{Adjusted} = .02$	$F(2, 492) = 67.3$, $p = .007$, $R^2_{Adjusted} = .21$
Age	**-0.002114*** **	**-0.006898*** **	**-0.005400*** **	-0.000342	**0.001298*** **
Sex	**0.026582*** **	**0.063142*** **	**0.047175*** **	**-0.017257** **	-0.002542

12

Supplemental Table 2: Tractwise age effects and sex differences on morphometry (length, Volume, and VLR) and microstructure (FA and MD) while modeling the interaction by sex on age: list of regression coefficients. All models are multivariate linear regressions based on baseline-normalized measures (x_{perc}) with overall F-statistics, *p*-values, and effect sizes ($R^2_{adjusted}$) listed. Slopes are noted below each regression equation and significant associations bolded with asterisks indicating significance. (*:p<.05, **:p<.01, ***:p<.001), with positive coefficients indicating M>F for sex differences.

	**Morphometry**			**Microstructure**	
Tract	Length$_{perc}$	Volume$_{perc}$	VLR$_{perc}$	FA$_{perc}$	MD$_{perc}$
**Fmajor**	$F(3, 489) = 8.43$, $p < .001$, $R^2_{Adjusted} = .04$	$F(3, 489) = 42.7$, $p < .001$, $R^2_{Adjusted} = .20$	$F(3, 489) = 54.9$, $p < .001$, $R^2_{Adjusted} = .25$	$F(3, 489) = 8.66$, $p < .001$, $R^2_{Adjusted} = .04$	$F(3, 489) = 29.8$, $p < .001$, $R^2_{Adjusted} = .15$
Age	-0.000042	**-0.004438*** **	**-0.004380*** **	**-0.0005326***	**0.001139*** **
Sex	0.017716	**0.156400***	**0.150016***	-0.0186586	0.011634
Age*Sex	-0.000658	-0.000264	0.000261	-0.0000165	-0.000414
**Fminor**	$F(3, 489) = 34.6$, $p < .001$, $R^2_{Adjusted} = .17$	$F(3, 489) = 81.7$, $p < .001$, $R^2_{Adjusted} = .33$	$F(3, 489) = 77.7$, $p < .001$, $R^2_{Adjusted} = .32$	$F(3, 489) = 24.2$, $p < .001$, $R^2_{Adjusted} = .12$	$F(3, 489) = 70.8$, $p < .001$, $R^2_{Adjusted} = .30$
Age	**-0.001797*** **	**-0.007734*** **	**-0.006512*** **	**-0.001716*** **	**0.002259*** **

Sex	-0.007020	**-0.224279*** **	**-0.213792*** **	0.003332	**0.038144* **
Age*Sex	0.000244	0.002040	0.001722	0.000259	**-0.000760** **
**ATR**	*F*(3, 493) = 4.52, p = .004, R^2_{Adjusted} = .02	*F*(3, 493) = 13.2, p < .001, R^2_{Adjusted} = .07	*F*(3, 493) = 14.1, p < .001, R^2_{Adjusted} = .07	*F*(3, 493) = 4.37, p = .005, R^2_{Adjusted} = .02	*F*(3, 493) = 66.9, p < .001, R^2_{Adjusted} = .29
Age	-0.000212	**-0.00278*** **	**-0.002648*** **	**-0.000936*** **	**0.002006*** **
Sex	0.024466	0.10013	0.075301	**-0.053971* **	**0.031352* **
Age*Sex	-0.000174	-0.00171	-0.001547	**0.001060* **	**-0.000670*** **
**CAB**	*F*(3, 486) = 29.0, p < .001, R^2_{Adjusted} = .15	*F*(3, 486) = 18.6, p < .001, R^2_{Adjusted} = .10	*F*(3, 486) = 13.8, p < .001, R^2_{Adjusted} = .07	*F*(3, 486) = 7.96, p < .001, R^2_{Adjusted} = .04	*F*(3, 486) = 11.5, p < .001, R^2_{Adjusted} = .06
Age	**-0.002092*** **	**-0.00396*** **	**-0.002399* **	**-0.001830** **	**0.000888*** **
Sex	0.059210	**0.33907** **	**0.294336** **	-0.015239	**0.032392* **
Age*Sex	-0.000926	**-0.00472** **	**-0.003969* **	0.000107	**-0.000511* **
**CCG**	*F*(3, 491) = 3.96, p = .008, R^2_{Adjusted} = .02	*F*(3, 491) = 11.9, p < .001, R^2_{Adjusted} = .06	*F*(3, 491) = 12.0, p < .001, R^2_{Adjusted} = .06	*F*(3, 491) = 14.9, p < .001, R^2_{Adjusted} = .08	*F*(3, 491) = 40.1, p < .001, R^2_{Adjusted} = .19
Age	-0.0001883	**-0.004051*** **	**-0.003997*** **	**-0.002118*** **	**0.001417*** **
Sex	-0.0098067	-0.052369	-0.045200	-0.017189	0.018543
Age*Sex	-0.0000963	0.000361	0.000506	0.000694	-0.000386
**CST**	*F*(3, 487) = 9.34, p < .001, R^2_{Adjusted} = .05	*F*(3, 487) = 16.0, p < .001, R^2_{Adjusted} = .08	*F*(3, 487) = 15.9, p < .001, R^2_{Adjusted} = .08	*F*(3, 487) = 0.78, p = .508, R^2_{Adjusted} = -.001	*F*(3, 487) = 9.22, p < .001, R^2_{Adjusted} = .05
Age	**-0.000738*** **	**-0.001309* **	-0.00061	-0.000284	**0.000590*** **
Sex	-0.011030	-0.004978	0.01229	-0.026594	-0.005025
Age*Sex	0.000288	-0.000959	-0.00130	0.000422	-0.000167
**ILF**	*F*(3, 493) = 8.11, p < .001, R^2_{Adjusted} = .04	*F*(3, 493) = 21.7, p < .001, R^2_{Adjusted} = .11	*F*(3, 493) = 19.2, p < .001, R^2_{Adjusted} = .10	*F*(3, 493) = 11.0, p < .001, R^2_{Adjusted} = .06	*F*(3, 493) = 44.0, p < .001, R^2_{Adjusted} = .21
Age	**-0.000761*** **	**-0.004605*** **	**-0.003998*** **	**-0.001217*** **	**0.001590*** **
Sex	-0.001362	0.009003	0.004396	-0.037818	0.016280
Age*Sex	0.000229	-0.000156	-0.000245	0.000466	-0.000505
**SLFP**	*F*(3, 493) = 32.3, p < .001, R^2_{Adjusted} = .16	*F*(3, 493) = 24.0, p < .001, R^2_{Adjusted} = .12	*F*(3, 493) = 16.2, p < .001, R^2_{Adjusted} = .08	*F*(3, 493) = 6.03, p < .001, R^2_{Adjusted} = .03	*F*(3, 493) = 47.8, p < .001, R^2_{Adjusted} = .22
Age	**-0.001466*** **	**-0.00400*** **	**-0.002784*** **	**-0.001018*** **	**0.001702*** **

Sex	0.033445	**0.24355****	**0.214256****	**-0.051273***	0.018511
Age*Sex	-0.000700	**-0.00350***	**-0.002947***	**0.001102****	-0.000484
**SLFT**	*F*(3, 491) = 5.16, *p* = .002, R^2_{Adjusted} = .02	*F*(3, 491) = 36.5, *p* < .001, R^2_{Adjusted} = .18	*F*(3, 491) = 36.2, *p* < .001, R^2_{Adjusted} = .18	*F*(3, 491) = 5.6, *p* < .001, R^2_{Adjusted} = .03	*F*(3, 491) = 51.7, *p* < .001, R^2_{Adjusted} = .24
Age	**-0.0004895***	**-0.001817***	**-0.001486***	**-0.000672****	**0.001759*****
Sex	0.0175363	**0.332798*****	**0.305583*****	**-0.060134****	0.019714
Age*Sex	-0.0000717	**-0.002799***	**-0.002565***	**0.001258****	-0.000480
**UNC**	*F*(3, 491) = 27.4, *p* < .001, R^2_{Adjusted} = .14	*F*(3, 491) = 57.6, *p* < .001, R^2_{Adjusted} = .26	*F*(3, 491) = 54.3, *p* < .001, R^2_{Adjusted} = .25	*F*(3, 491) = 3.43, *p* = .017, R^2_{Adjusted} = .01	*F*(3, 491) = 47.3, *p* < .001, R^2_{Adjusted} = .22
Age	**-0.002393*****	**-0.0069222*****	**-0.005268*****	-0.000422	**0.001521*****
Sex	-0.013894	0.0596197	0.066327	-0.028965	**0.029703***
Age*Sex	0.000695	0.0000605	-0.000329	0.000201	**-0.000554***

Supplemental Table 3: Tractwise linear and quadratic age effects on morphometry (length, Volume, and VLR) and microstructure (FA and MD): list of regression coefficients.
All models are multivariate linear regressions based on baseline-normalized measures (x_{perc}) with overall F-statistics, *p*-values, and effect sizes ($R^2_{adjusted}$) listed. Slopes are noted below each regression equation and significant associations bolded with asterisks indicating significance. (*:p<.05, **:p<.01, ***:p<.001).

	**Morphometry**			**Microstructure**	
Tract	Length_{perc}	Volume_{perc}	VLR_{perc}	FA_{perc}	MD_{perc}
**Fmajor (linear)**	*F*(1, 491) = 4.12, *p* = .043, R^2_{Adjusted} = .006	*F*(1, 491) = 49.0, *p* < .001, R^2_{Adjusted} = .09	*F*(1, 491) = 43.9, *p* < .001, R^2_{Adjusted} = .08	*F*(1, 491) = 10.7, *p* = .001, R^2_{Adjusted} = .02	*F*(1, 491) = 69.1, *p* = .001, R^2_{Adjusted} = .12
Age	**-0.000353***	**-0.004272*****	**-0.003952*****	**-0.000578****	**0.000944*****
**Fmajor (quadr.)**	*F*(1, 491) = 4.30, *p* = .039, R^2_{Adjusted} = .007	*F*(1, 491) = 52.3, *p* < .001, R^2_{Adjusted} = .09	*F*(1, 491) = 47.1, *p* < .001, R^2_{Adjusted} = .09	*F*(1, 491) = 9.64, *p* = .002, R^2_{Adjusted} = .02	*F*(1, 491) = 76.4, *p* < .001, R^2_{Adjusted} = .13
Age^2	**-0.00000299***	**-0.00003646*****	**-0.00003379*****	**-0.00000455****	**0.000008163*****
**Fminor (linear)**	*F*(1, 491) = 101, *p* < .001, R^2_{Adjusted} = .17	*F*(1, 491) = 174, *p* < .001, R^2_{Adjusted} = .26	*F*(1, 491) = 143, *p* < .001, R^2_{Adjusted} = .22	*F*(1, 491) = 61.3, *p* < .001, R^2_{Adjusted} = .12	*F*(1, 491) = 199, *p* < .001, R^2_{Adjusted} = .29
Age	**-0.001684*****	**-0.007108*****	**-0.006029*****	**-0.001577*****	**0.001940*****
**Fminor (quadr.)**	*F*(1, 491) = 105, *p* < .001, R^2_{Adjusted} = .17	*F*(1, 491) = 177, *p* < .001, R^2_{Adjusted} = .26	*F*(1, 491) = 145, *p* < .001, R^2_{Adjusted} = .23	*F*(1, 491) = 55.3, *p* < .001, R^2_{Adjusted} = .10	*F*(1, 491) = 209, *p* < .001, R^2_{Adjusted} = .30

Age^2	**-0.00001416*** **	**-0.00005913*** **	**-0.00005014*** **	**-0.00001246*** **	**0.00001633*** **
**ATR (linear)**	$F(1, 495) = 2.76$, $p = .097$, $R^2_{Adjusted} = .004$	$F(1, 495) = 37.5$, $p < .001$, $R^2_{Adjusted} = .07$	$F(1, 495) = 39.3$, $< .001$, $R^2_{Adjusted} = .07$	$F(1, 495) = 5.33$, $= .021$, $R^2_{Adjusted} = .009$	$F(1, 495) = 186$, $< .001$, $R^2_{Adjusted} = .27$
Age	-0.000255	**-0.003477*** **	**-0.003310*** **	**-0.000487* **	**0.001717*** **
**ATR (quadr.)**	$F(1, 495) = 3.78$, $p = .052$, $R^2_{Adjusted} = .006$	$F(1, 495) = 41.5$, $p < .001$, $R^2_{Adjusted} = .08$	$F(1, 495) = 42.9$, $p < .001$, $R^2_{Adjusted} = .08$	$F(1, 495) = 4.79$, $p = .029$, $R^2_{Adjusted} = .008$	$F(1, 495) = 206$, $p < .001$, $R^2_{Adjusted} = .29$
Age^2	-0.00000248	**-0.00003020*** **	**-0.00002856*** **	**-0.00000383* **	**0.00001475*** **
**CAB (linear)**	$F(1, 488) = 83.4$, $p < .001$, $R^2_{Adjusted} = .14$	$F(1, 488) = 41.7$, $p < .001$, $R^2_{Adjusted} = .08$	$F(1, 488) = 26.0$, $p < .001$, $R^2_{Adjusted} = .05$	$F(1, 488) = 23.2$, $p < .001$, $R^2_{Adjusted} = .04$	$F(1, 488) = 29.8$, $p < .001$, $R^2_{Adjusted} = .06$
Age	**-0.002459*** **	**-0.005767*** **	**-0.003903*** **	**-0.001802*** **	**0.000685*** **
**CAB (quadr.)**	$F(1, 488) = 92.2$, $p < .001$, $R^2_{Adjusted} = .16$	$F(1, 488) = 44.3$, $p < .001$, $R^2_{Adjusted} = .08$	$F(1, 488) = 27.5$, $p < .001$, $R^2_{Adjusted} = .05$	$F(1, 488) = 23.5$, $p < .001$, $R^2_{Adjusted} = .04$	$F(1, 488) = 32.3$, $p < .001$, $R^2_{Adjusted} = .06$
Age^2	**-0.00002121*** **	**-0.00004902*** **	**-0.00003312*** **	**-0.00001498*** **	**0.00000588*** **
**CCG (linear)**	$F(1, 493) = 2.19$, $p = .139$, $R^2_{Adjusted} = .002$	$F(1, 493) = 33.3$, $p < .001$, $R^2_{Adjusted} = .06$	$F(1, 493) = 35.3$, $p < .001$, $R^2_{Adjusted} = .07$	$F(1, 493) = 35.8$, $p < .001$, $R^2_{Adjusted} = .07$	$F(1, 493) = 116$, $p < .001$, $R^2_{Adjusted} = .19$
Age	-0.000254	**-0.003961*** **	**-0.003821*** **	**-0.0018*** **	**0.001254*** **
**CCG (quadr.)**	$F(1, 493) = 3.07$, $p = .080$, $R^2_{Adjusted} = .004$	$F(1, 493) = 36.7$, $p < .001$, $R^2_{Adjusted} = .07$	$F(1, 493) = 38.2$, $p < .001$, $R^2_{Adjusted} = .07$	$F(1, 493) = 31.9$, $p < .001$, $R^2_{Adjusted} = .06$	$F(1, 493) = 127$, $p < .001$, $R^2_{Adjusted} = .20$
Age^2	-0.00000249	**-0.00003432*** **	**-0.00003282*** **	**-0.00001408*** **	**0.000010772*** **
**CST (linear)**	$F(1, 489) = 24.1$, $p < .001$, $R^2_{Adjusted} = .05$	$F(1, 489) = 19.1$, $p < .001$, $R^2_{Adjusted} = .04$	$F(1, 489) = 10.4$, $p = .001$, $R^2_{Adjusted} = .02$	$F(1, 489) = 21.4$, $p = .001$, $R^2_{Adjusted} = .08$	$F(1, 489) = 13.0$, $p < .001$, $R^2_{Adjusted} = .02$
Age	**-0.000611*** **	**-0.001815*** **	**-0.00126** **	-0.000117	**0.000494*** **
**CST (quadr.)**	$F(1, 489) = 24.7$, $p < .001$, $R^2_{Adjusted} = .05$	$F(1, 489) = 20.7$, $p < .001$, $R^2_{Adjusted} = .04$	$F(1, 489) = 11.6$, $p < .001$, $R^2_{Adjusted} = .02$	$F(1, 489) = 0.24$, $p = .623$, $R^2_{Adjusted} = -.002$	$F(1, 489) = 15.2$, $p < .001$, $R^2_{Adjusted} =.03$
Age^2	**-0.00000511*** **	**-0.00001563*** **	**-0.00001101*** **	-0.000000661	**0.00000442*** **
**ILF (linear)**	$F(1, 495) = 16.9$, $p < .001$, $R^2_{Adjusted} = .03$	$F(1, 495) = 65.4$, $p < .001$, $R^2_{Adjusted} = .12$	$F(1, 495) = 57.2$, $p < .001$, $R^2_{Adjusted} = .10$	$F(1, 495) = 28.1$, $p < .001$, $R^2_{Adjusted} = .05$	$F(1, 495) = 112$, $p < .001$, $R^2_{Adjusted} = .18$
Age	**-0.000648*** **	**-0.004669*** **	**-0.004115*** **	**-0.001046*** **	**0.001362*** **

**ILF (quadr.)**	$F(1, 495) = 18.7$, $p <$ .001, $R^2_{Adjusted} = .03$	$F(1, 495) = 72.4$, $p <$ .001, $R^2_{Adjusted} = .13$	$F(1, 495) = 63.3$, $p <$ .001, $R^2_{Adjusted} = .11$	$F(1, 495) = 26.8$, $p <$ .001, $R^2_{Adjusted} = .05$	$F(1, 495) = 119$, $p <$ .001, $R^2_{Adjusted} = .19$
Age^2	**-0.00000564*** **	**-0.00004040*** **	**-0.00003562*** **	**-0.00000846*** **	**0.00001155*** **
**SLFP (linear)**	$F(1, 495) = 90.9$, $p <$ .001, $R^2_{Adjusted} = .15$	$F(1, 495) = 60.5$, $p <$ .001, $R^2_{Adjusted} = .12$	$F(1, 495) = 37.4$, $p <$ .001, $R^2_{Adjusted} = .07$	$F(1, 495) = 6.70$, $p =$ .010, $R^2_{Adjusted} = .01$	$F(1, 495) = 131$, $p <$ .001, $R^2_{Adjusted} = .21$
Age	**-0.001766*** **	**-0.005357*** **	**-0.00391*** **	**-0.000542** **	**0.00148*** **
**SLFP (quadr.)**	$F(1, 495) = 104$, $p <$ .001, $R^2_{Adjusted} = .17$	$F(1, 495) = 69.2$, $p <$ .001, $R^2_{Adjusted} = .12$	$F(1, 495) = 42.8$, $p <$ .001, $R^2_{Adjusted} = .08$	$F(1, 495) = 6.03$, $p =$ .014, $R^2_{Adjusted} = .01$	$F(1, 495) = 140$, $p <$ .001, $R^2_{Adjusted} = .22$
Age^2	**-0.00001544*** **	**-0.00004707*** **	**-0.00003449*** **	**-0.00000426* **	**0.00001262*** **
**SLFT (linear)**	$F(1, 493) = 8.25$, $p =$ .004, $R^2_{Adjusted} = .01$	$F(1, 493) = 15.5$, $p <$ .001, $R^2_{Adjusted} = .03$	$F(1, 493) = 13.4$, $p <$ .001, $R^2_{Adjusted} = .02$	$F(1, 493) = 0.53$, $p =$ .456, $R^2_{Adjusted} = -.001$	$F(1, 493) = 145$, $p <$ .001, $R^2_{Adjusted} = .23$
Age	**-0.000498** **	**-0.002691*** **	**-0.002286*** **	-0.000140	**0.001551*** **
**SLFT (quadr.)**	$F(1, 493) = 7.94$, $p =$ .005, $R^2_{Adjusted} = .01$	$F(1, 493) = 17.1$, $p <$ .001, $R^2_{Adjusted} = .03$	$F(1, 493) = 15.2$, $p <$ .001, $R^2_{Adjusted} = .03$	$F(1, 493) = 0.30$, $p =$ .584, $R^2_{Adjusted} = .-001$	$F(1, 493) = 155$, $p <$ .001, $R^2_{Adjusted} = .24$
Age^2	**-0.00000404** **	**-0.00002338*** **	**-0.00002010*** **	-0.000000869	**0.00001316*** **
**UNC (linear)**	$F(1, 493) = 65.5$, $p <$ .001, $R^2_{Adjusted} = .12$	$F(1, 493) = 152$, $p <$ .001, $R^2_{Adjusted} = .24$	$F(1, 493) = 145$, $p <$ .001, $R^2_{Adjusted} = .23$	$F(1, 493) = 2.83$, $p =$ .093, $R^2_{Adjusted} = .004$	$F(1, 493) = 134$, $p <$ .001, $R^2_{Adjusted} = .21$
Age	**-0.002067*** **	**-0.00679*** **	**-0.005317*** **	-0.000372	**0.001294*** **
**UNC (quadr.)**	$F(1, 493) = 67.0$, $p <$ .001, $R^2_{Adjusted} = .12$	$F(1, 493) = 162$, $p <$ .001, $R^2_{Adjusted} = .25$	$F(1, 493) = 158$, $p <$ .001, $R^2_{Adjusted} = .24$	$F(1, 493) = 2.27$, $p =$ .133, $R^2_{Adjusted} = .003$	$F(1, 493) = 146$, $p <$ .001, $R^2_{Adjusted} = .23$
Age^2	**-0.00001728*** **	**-0.00005748*** **	**-0.00004541*** **	-0.00000276	**0.000011080*** **

Supplemental Table 4: Tractwise cross-correlations between morphometry (length and Volume) and microstructure (FA, AD, and RD): list of correlation values.

	Fmajor	Fminor	ATR	CAB	CCG	CST	ILF	SLFP	SLFT	UNC
Len-FA		r(491) = .24, p<.001								
Len-AD		r(491) = .34, p<.001	r(495) = .12, p=.006	r(488) = .13, p=.002			r(495) = .18, p<.001	r(495) = .34, p<.001		
Len-RD		r(491) = .24, p<.001	r(495) = .11, p=.009				r(495) = .17, p<.001	r(495) = .23, p<.001		
Vol-FA					r(493) = .12, p=.006				r(493) = .15, p<.001	r(493) = .20, p<.001
Vol-AD	r(491) = .14, p=.002		r(495) = .19, p<.001		r(493) = .17, p<.001	r(489) = .17, p<.001	r(495) = .25, p<.001	r(495) = .33, p<.001	r(493) = .16, p<.001	r(493) = .34, p<.001
Vol-RD			r(495) = .19, p<.001				r(495) = .22, p<.001	r(495) = .20, p<.001		
AD-RD		r(491) = .53, p<.001	r(495) = .76, p<.001				r(495) = .38, p<.001	r(495) = .33, p<.001		
AD-FA		r(491) = .24, p<.001			r(493) = .14, p=.002					
RD-FA		r(491) = .54, p<.001								
Len-Vol			r(495) = .41, p<.001	r(488) = .67, p<.001	r(493) = .39, p<.001		r(495) = .40, p<.001	r(495) = .50, p<.001		
